# The intracellular plasma membrane-connected compartment in the assembly of HIV-1 in human macrophages

**DOI:** 10.1186/s12915-016-0272-3

**Published:** 2016-06-23

**Authors:** David O. Nkwe, Annegret Pelchen-Matthews, Jemima J. Burden, Lucy M. Collinson, Mark Marsh

**Affiliations:** MRC Laboratory for Molecular Cell Biology, University College London, Gower Street, London, WC1E 6BT UK; The Francis Crick Institute, Lincoln’s Inn Fields Laboratories, 44 Lincoln’s Inn Fields, London, WC2A 3LY UK; Present Address: Department of Biology and Biotechnological Sciences, College of Science, Botswana International University of Science and Technology, Private Bag 16, Palapye, Botswana

**Keywords:** HIV-1, Assembly compartment, Macrophage, Intracellular plasma membrane-connected compartments, Endosomal sorting complexes required for transport, Volume electron microscopy

## Abstract

**Background:**

In HIV-infected macrophages, newly formed progeny virus particles accumulate in intracellular plasma membrane-connected compartments (IPMCs). Although the virus is usually seen in these compartments, it is unclear whether HIV assembly is specifically targeted to IPMCs or whether some viruses may also form at the cell surface but are not detected, as particles budding from the latter site will be released into the medium.

**Results:**

To investigate the fidelity of HIV-1 targeting to IPMCs compared to the cell surface directly, we generated mutants defective in recruitment of the Endosomal Sorting Complexes Required for Transport (ESCRT) proteins required for virus scission. For mutants unable to bind the ESCRT-I component Tsg101, HIV release was inhibited and light and electron microscopy revealed that budding was arrested. When expressed in human monocyte-derived macrophages (MDM), these mutants formed budding-arrested, immature particles at their assembly sites, allowing us to capture virtually all of the virus budding events. A detailed morphological analysis of the distribution of the arrested viruses by immunofluorescence staining and confocal microscopy, and by electron microscopy, demonstrated that HIV assembly in MDMs is targeted primarily to IPMCs, with fewer than 5 % of budding events seen at the cell surface. Morphometric analysis of the relative membrane areas at the cell surface and IPMCs confirmed a large enrichment of virus assembly events in IPMCs.

Serial block-face scanning electron microscopy of macrophages infected with a budding-defective HIV mutant revealed high-resolution 3D views of the complex organisation of IPMCs, with in excess of 15,000 associated HIV budding sites, and multiple connections between IPMCs and the cell surface.

**Conclusions:**

Using detailed quantitative analysis, we demonstrate that HIV assembly in MDMs is specifically targeted to IPMCs. Furthermore, 3D analysis shows, for the first time, the detailed ultrastructure of an IPMC within a large cell volume, at a resolution that allowed identification of individual virus assembly events, and potential portals through which virus may be released during cell-cell transfer. These studies provide new insights to the organisation of the HIV assembly compartments in macrophages, and show how HIV particles accumulating in these protected sites may function as a virus reservoir.

**Electronic supplementary material:**

The online version of this article (doi:10.1186/s12915-016-0272-3) contains supplementary material, which is available to authorized users.

## Background

HIV primarily infects activated T cells, in particular effector memory T cells, and macrophages expressing CD4 and the co-receptor CCR5 (Reviewed in [1–3]). Subsequently, following replication, progeny viruses are produced through the assembly of the main viral components at the plasma membrane of infected cells. A key difference in HIV assembly in these different host cells is that, in mature macrophages, newly formed virions accumulate in characteristic highly interconnected intracellular compartments [4–9]. Many of these compartments have been shown to be connected to the plasma membrane, so that they are now known as intracellular plasma membrane-connected compartments (IPMCs). The compartments contain various markers typical of the plasma membrane, including proteins such as CD44, the tetraspanins CD9, CD81 or CD53 [4, 10], as well as β2 integrins (CD18 with CD11b and/or CD11c) and associated proteins that can form focal adhesion-like structures [7, 9] or the scavenger receptor CD36 [11]. In addition, the plasma membrane and IPMC membranes both contain cholesterol and phosphatidylinositol-4,5-bisphosphate [PtdIns(4,5)P_2_] [3, 9]. Similar compartments are present in differentiated/mature uninfected monocyte-derived macrophages (MDMs), but are expanded upon HIV infection [4, 7, 10, 11]. In addition, similar compartments have been seen in lymph node macrophages in vivo [12] or in isolated placental Hoffbauer cells [13], and appear also to be present in gut-associated lymphoid tissue of HIV-infected humanized mice [14]. Notably, when HIV-infected MDMs interact with autologous CD4^+^ T cells, viruses contained within IPMCs can be released through virological synapses leading to efficient infection of the T cells [15–18]. Macrophages become prominent target cells of HIV in advanced disease and AIDS, when CD4^+^ T cells are depleted [19]. In particular, HIV-infected monocytes invading the brain differentiate to macrophages that mediate neuroinflammation and contribute to pathogenesis and HIV-associated neurocognitive disorders (reviewed in [20–22]). Furthermore, these cells may represent a viral reservoir at this immunologically privileged site [23, 24].

HIV assembly is targeted to the plasma membrane via PtdIns(4,5)P_2_, which binds the Matrix (MA) domain of the main virally encoded structural protein p55Gag [25–27]. As PtdIns(4,5)P_2_ has been shown to be present in IPMCs, as well as at the cell surface proper, both locations should be able to support virion formation. However, the fate of viruses budding at the cell surface or within IPMCs would differ, in that viruses assembling at the cell surface would be released into the extracellular fluids and may be able to infect other distant cells via cell-free infection. By contrast, viruses that accumulate in IPMCs could be stored in an infectious form for extended periods of time [28] and may be a source of virus for direct cell-to-cell transmission to non-infected target cells. Despite these different viral fates, to date, there has not been a rigorous analysis to address whether, in infected macrophages, HIV assembles primarily within IPMCs or whether a proportion assembles at the cell surface and is released directly into the medium.

Herein, we have addressed this question by generating HIV-1 mutants defective in the recruitment of Endosomal Sorting Complexes Required for Transport (ESCRT) proteins required for virus scission [29, 30] (reviewed in [31]). When expressed in MDMs, these mutant viruses formed immature virus buds that were arrested at their budding sites, allowing the visualisation of all virus assembly events. Detailed analysis of HIV assembly sites by confocal imaging and by electron microscopy (EM) demonstrated that HIV assembly in MDMs is targeted primarily to the IPMCs. Furthermore, using serial block-face scanning EM of MDMs infected with a budding-defective HIV mutant, we constructed a high-resolution 3-dimensional (3D) model of the complex organisation of the IPMC in relation to the cell surface and mapped the associated HIV budding sites. These studies indicate that, in infected mature MDMs, HIV assembly is targeted to IPMCs, provide a deeper understanding of the organisation of the HIV assembly compartments in MDMs, and show how infected macrophages accumulating HIV particles in these protected sites may function as a virus reservoir.

## Results

### Generation of budding-arrested HIV-1 proviruses

HIV requires the ESCRT machinery to complete budding and release free virions [31–34]. Thus, we exploited the ability to inhibit recruitment of the ESCRT machinery to arrest virus budding and allow analysis of all the sites where virus assembly is targeted in MDMs. Although ESCRT components, such as Tsg101 (tumour susceptibility gene 101) and/or ALIX (ALG-2-interacting protein X), were efficiently depleted by siRNA, we observed little effect on virus release (Additional file 1: Figure S1), which probably reflects the difficulty of achieving effective knockdown of the ESCRT proteins in the cells infected with HIV.

**Fig. 1:**
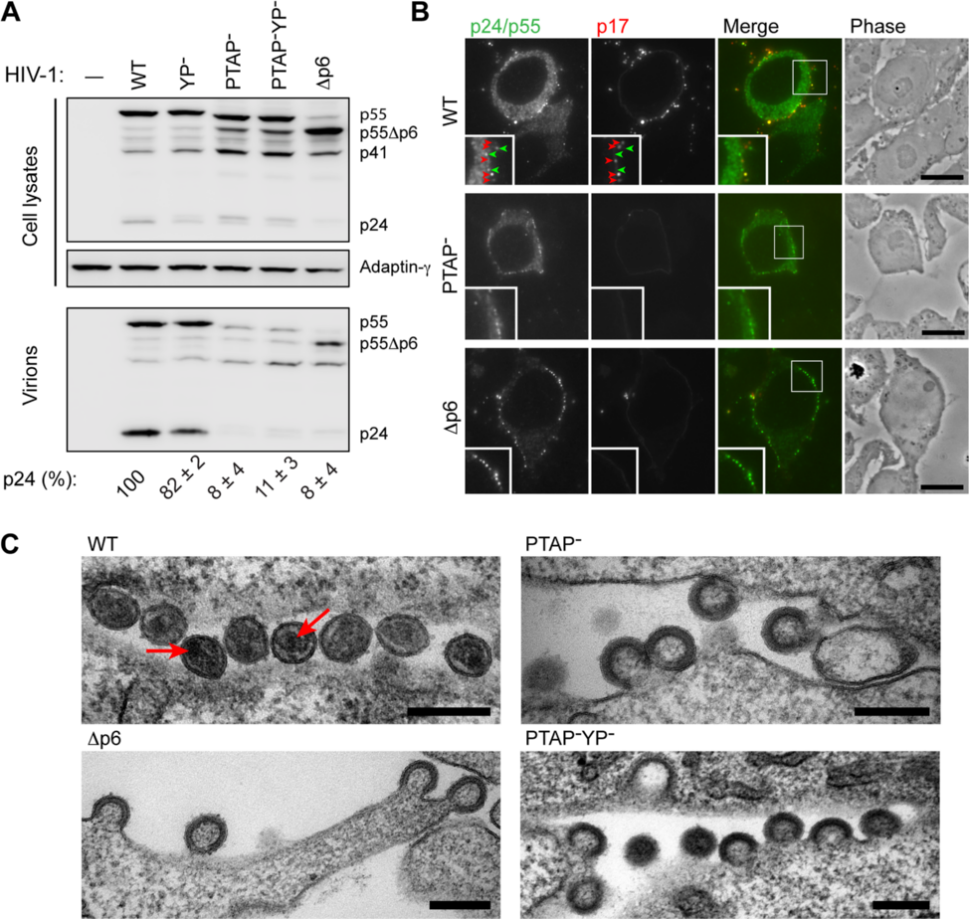
Characterisation of HIV-1 R3A p6 domain mutants in HEK 293 T cells. HEK 293 T cells were transfected with the indicated HIV-1 pNL4.3-R3A constructs (see Additional file 2: Figure S2A), and cultured for 24 h before analysis. **a** Western blot of cell lysates and released virions purified from the media. Blots were probed with antibodies against viral p24/55 or adaptin-γ as a loading control. The positions of p55Gag, Gag protein lacking the p6 domain (p55Δp6), the p41 cleavage product and mature p24 are indicated. Band signal intensities were quantified using ImageJ to determine the virus release efficiencies shown below the blot (data from two independent experiments ± standard deviation). **b** Immunofluorescence staining of semi-thin cryo-sections (0.5 μm) with antibodies against p24/55 (38:96 K and EF7, *green*) and p17 (4C9, *red*). Bright puncta of p24/p55 (e.g. at the *green arrowheads*) indicate assembling viruses. For the R3A WT, some mature cell surface puncta were labelled for p17 (*red arrowheads*) suggesting they are mature virions, but for the PTAP^–^ and Δp6 mutants, the cell surface contained only p24/p55-labelled puncta (*green*) devoid of p17 staining, indicative of immature virus buds. Scale bars, 10 μm. **c** Ultrastructure of HEK 293 T cells expressing the indicated HIV-1 R3A constructs. Transfected cells were embedded in Epon and processed for electron microscopy. Cells expressing the HIV-1 R3A WT contained rare mature virus particles such as the group of particles trapped between adjacent cells shown here. *Arrows* mark electron-dense cores visible in some of the particles. In contrast, only immature budding-arrested virus particles were seen on cells expressing the PTAP^–^, PTAP^–^YP^–^ and Δp6 mutant proviruses. Scale bars, 200 nm

As an alternative approach, we generated full-length release-defective HIV-1 R3A proviral clones in which the ESCRT interacting motifs were disrupted. Specifically, the PTAP motif in the p6 domain of Gag, which is required to recruit Tsg101, was mutated to LIRL ([29], hereafter referred to as PTAP^–^), or the first two amino acids of the consensus sequence for ALIX interaction, YPLASL, were changed to SR ([30], hereafter referred to as YP^–^), or these mutations were combined in a PTAP^–^YP^–^ provirus. Alternatively, the entire p6 domain was deleted to generate the Δp6 mutant (Additional file 2: Figure S2). To verify that these mutants were budding arrested, the proviral constructs were transfected into HEK 293 T cells and the media and cell lysates analysed for released virions and viral proteins, respectively, by SDS-PAGE and western blotting (Fig. 1a). Analysis of cell lysates revealed that similar amounts of the Gag polyproteins were expressed for all proviruses, with the expected molecular weights; the Δp6 Gag migrated at about 49 kDa, while PTAP^–^ containing mutants showed the characteristic mobility shift for p55Gag and increased levels of the processing intermediate p41 (representing a matrix-capsid fragment), as has been described previously [29, 35]. Analysis of released particles in the media showed a strong decrease in both p55Gag and the processed capsid protein p24 in media from cells transfected with the PTAP^–^, PTAP^–^YP^–^ and Δp6 proviruses. The presence of p55Gag in the culture supernatants is indicative of immature viruses, but may also arise from sheared immature particles, microvesicles or cell debris. The nearly complete absence of mature processed p24 in supernatants from the PTAP^–^, PTAP^–^YP^–^ and Δp6 viruses suggests that bone fide budding of progeny virions is arrested.

Similar cultures of HEK 293 T cells were processed for morphological analysis either by cryosectioning and immunolabelling, or embedding in Epon for EM. Analysis of semi-thin immunolabelled cryosections by fluorescence microscopy showed frequent infected cells, identified by cytoplasmic p24/p55 Gag labelling (Fig. 1b). Assembling virus particles appeared as brightly stained puncta at the cell surface (see p24/p55 panels, green). Notably, for cells transfected with HIV-1 R3A wild type (WT), some of these puncta could be co-labelled with an antibody (mAb 4C9) that is specific for the proteolytically cleaved MA protein p17 (red), indicating that they are mature or maturing virions. While p55-stained cell surface particles were also seen for cells transfected with the PTAP^–^ or Δp6 proviruses, consistent with virus budding, these failed to co-label for p17, indicating that they had not undergone maturation. A budding arrested phenotype was directly confirmed by EM (Fig. 1c). Cells expressing R3A WT contained few virus particles, although some mature virions could be found, for example trapped between cells. For cells transfected with the mutant proviruses we only observed arrested buds with various degrees of curvature and lined with the thick Gag layer characteristic of immature HIV particles. This demonstrated that proviral clones unable to recruit Tsg101 (the PTAP^–^ and Δp6 mutants) were indeed arrested late during particle assembly, with incomplete, immature particles retained at their cell surface budding sites.

### Expression of budding-arrested HIV proviruses in primary MDMs

**Fig. 2:**
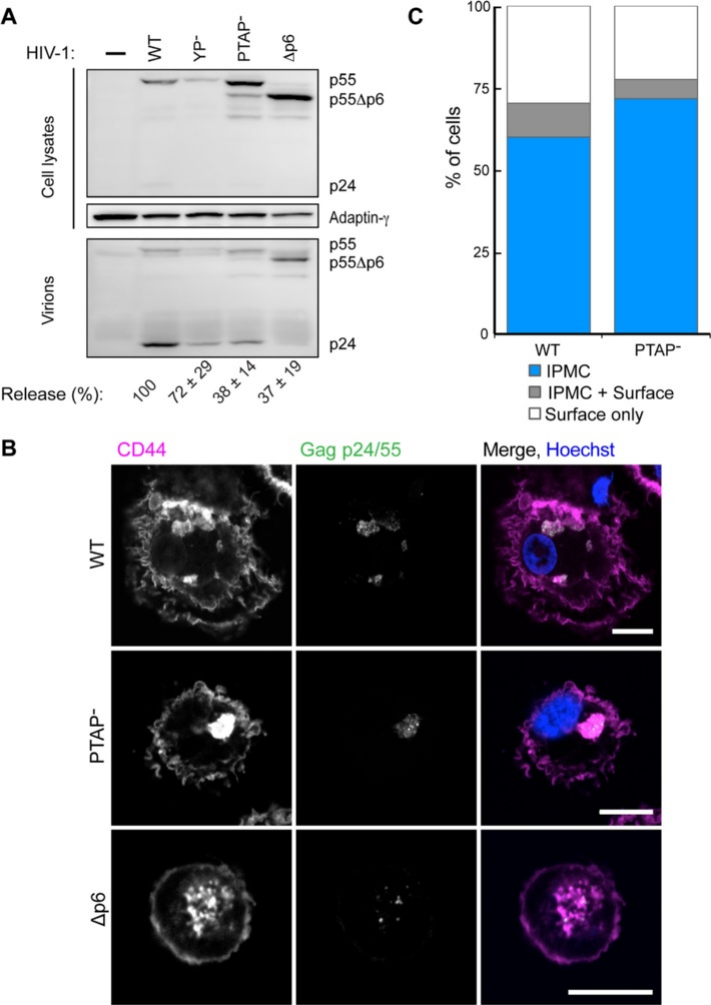
Transfection of monocyte-derived macrophages (MDMs) with HIV-1 R3A WT, PTAP^–^ or Δp6 mutants by electroporation. Fourteen-day-old MDMs were electroporated with the indicated provirus constructs and incubated for 24 h before analysis. **a** Released virions were collected and virus pellets and cells were lysed and analysed by western blotting as detailed in Fig. 1b, and signal intensities were quantified using ImageJ to determine the virus release efficiencies shown below the blot (data from three independent experiments ± standard deviation). **b** Cells were fixed and stained with antibodies to p24/55 (38:96 K and EF7, *green*) and CD44 (*magenta*). Representative single confocal sections from z-stack series are shown. Scale bars, 10 μm. **c** Distributions of virus staining on the immunolabelled MDMs (samples from three different blood donors). Coverslips were scanned systematically and virus-expressing cells scored into three categories of cells where virus was seen in the intracellular plasma membrane-connected compartments (IPMCs) only (*blue*), in IPMCs as well as the cell surface (*grey*), or only at the cell surface (*white*; these latter MDMs lacked CD44^+^ IPMCs)

To determine where these HIV mutants assemble in macrophages, monocytes isolated from donor buffy coats and differentiated to MDMs for 14 days were electroporated with the proviral clones and returned to culture for 24 h, when the cell lysates and released virions in the media were collected and analysed (Fig. 2a). Although the transfection efficiencies were low for these primary cells (<1 %), we did detect expression of the Gag polyproteins, with the lowest expression levels for the YP^–^ mutant. Analysis of the supernatants revealed robust release of HIV-1 R3A WT; by contrast, and as observed for transfected HEK 293 T cells, release of the PTAP^–^ or Δp6 proviruses was significantly reduced, indicating that completion of HIV budding is also ESCRT-dependent in macrophages.

Cultures of transfected MDMs were also fixed and immunolabelled with antibodies against the hyaluronic acid receptor CD44, a marker of the plasma membrane and IPMCs, and against Gag p24/p55, to identify infected cells and determine the location of the virus particles or buds, and analysed by confocal microscopy (Fig. 2b). For MDMs transfected with HIV-1 R3A WT, or the PTAP^–^ or Δp6 mutants, infected cells were seen in the cultures, and in all cases puncta of Gag p24/p55 staining were located primarily within CD44^+^ IPMC structures (Fig. 2b). Only a few cells expressed the Δp6 mutant, precluding any quantitative analysis. For the WT and PTAP^–^ transfections, coverslips were systematically searched and any Gag-expressing cells scored for whether Gag puncta were seen exclusively within CD44^+^ IPMCs, at the cell surface, or at both locations. This revealed that, in most MDMs, virus staining was only detected in the IPMCs (60 % and 72 % of cells, respectively, Fig. 2c), suggesting that virus assembly is targeted to this site. Notably, those cells in which virus staining was seen exclusively at the cell surface lacked detectable intracellular CD44^+^ IPMCs.

**Fig. 3:**
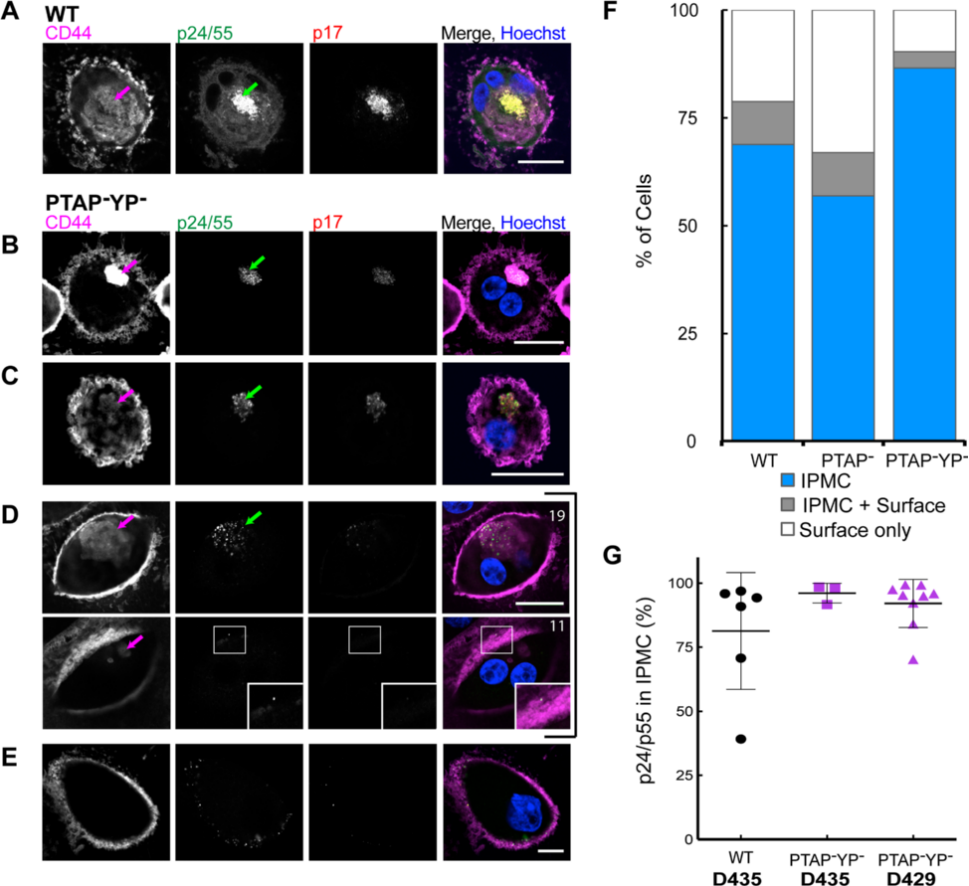
Distribution of HIV-1 R3A WT or the PTAP^–^YP^–^ and PTAP^–^ mutants in infected monocyte-derived macrophages (MDMs). Seven-day-old MDMs were infected with HIV-1 R3A WT or the rescued release-defective viruses HIV-1 R3A PTAP^−^YP^−^ or PTAP^−^ (see Additional file 2: Figure S2). Cultures were incubated for a further 7 days, fixed and immunolabelled with antibodies against p24/55 (Kal-1, *green*), p17 (4C9, *red*) and CD44 (*magenta*), and examined by confocal microscopy. **a**–**e** Selected single optical sections from z-stacks of MDMs infected with WT (**a**) or the PTAP^–^YP^–^ mutant (**b**–**e**, two different sections are shown for the cell in **d**). *Arrows* point out the intracellular plasma membrane-connected compartments (IPMCs). The boxed area in (**d**) is enlarged in the insets, revealing a single immature virus bud at the cell surface. Scale bars, 20 μm. **f** Quantitative analysis of virus distributions on MDMs from five different blood donors. Coverslips were scanned systematically and virus-expressing cells scored into three categories of cells where virus was seen in the IPMCs only (*blue*), in IPMCs as well as the cell surface (*grey*), or only at the cell surface (*white*; these latter MDMs lacked CD44^+^ IPMCs). **g** For selected cells with Gag p24/55 labelling both at the IPMC and the cell surface, the proportion of p24/p55 fluorescence in the IPMC was estimated and plotted. p24/p55Gag fluorescence was primarily in the IPMC for the WT (>80 %), and for cells from two donors infected with PTAP^−^YP^−^ mutant virus (>90 %). See also Additional file 3: Figure S3 and Additional file 4: Figure S4

To overcome the very low transfection efficiencies in primary MDMs and analyse HIV budding sites at higher resolution and more quantitatively, we prepared infectious stocks of the budding-arrested proviruses. HEK 293 T cells were co-transfected with the PTAP^–^ and PTAP^–^YP^–^ mutants together with pCMVGag, which expresses WT p55Gag, at a ratio of 12:1 (Additional file 2: Figure S2). This produced infectious virus stocks that could be used for single-cycle infections. MDMs were differentiated from monocytes for 7 days and then infected with HIV R3A WT or the rescued ESCRT mutant virus stocks and cultured for a further 7 days before fixation and analysis by immunofluorescence staining and confocal microscopy (Fig. 3 and Additional file 3: Figure S3 and Additional file 4: Figure S4). In MDM cultures infected with HIV-1 R3A WT, many cells had strong accumulations of viruses in IPMCs marked by CD44. Notably, these virus accumulations could be stained with antibodies against the capsid protein that react with both p55Gag and p24, as well as with the 4C9 antibody specific for the mature p17 matrix protein, indicating that these are mainly mature virus particles that have completed budding. For the mutants, p24/p55 staining was also seen within IPMC structures, but there was little reactivity with the p17 antibody, consistent with a budding-arrested phenotype (Fig. 3b, c and Additional file 3: Figure S3). On a few cells p24/p55 staining was seen in IPMCs as well as at the cell surface (Fig. 3d, Additional file 4: Figure S4A), while some infected MDMs lacked CD44-labelled IPMCs, and displayed p24/p55-stained virus puncta only at the cell surface (Fig. 3e and Additional file 3: Figure S3). To determine the distribution of the viruses and/or buds more quantitatively, we scored cells from five blood donors according to the distribution of the stained virus puncta (Fig. 3f). As for the transfected MDMs (Fig. 2), the majority of cells contained virus staining only in the IPMCs. Again, approximately 20–25 % of cells lacked IPMCs, but in cells where the compartments were present, virus assembly appeared to be targeted to these sites. Overall, in MDMs expressing HIV-1 R3A either after transfection (Fig. 2) or after infection with rescued virus stocks (Fig. 3), 66 % of cells infected with WT, or 77 % of cells with PTAP^–^ or PTAP^–^YP^–^ mutant viruses, only had virus staining in IPMCs. These observations suggest that HIV assembly is targeted specifically to the IPMCs.

For more quantitative analysis of the levels of viruses in infected cells, we recorded complete confocal z-stacks from random fields of infected MDMs, and measured the total fluorescence intensity for p24/55 and p17 staining of the infected cells (Additional file 4: Figure S4B and C, respectively). This confirmed strong labelling for viral p24/p55, though with somewhat stronger labelling of cells infected with WT virus compared to the PTAP^–^YP^–^ mutant (Additional file 4: Figure S4B). By contrast, strong labelling with the anti-p17 antibody specific for the cleaved MA was only seen for MDMs infected with R3A WT, while cells infected with the PTAP^–^YP^–^ mutant had rare p17-stained puncta, consistent with occasional events where HIV protease may have been activated prematurely in the bud [36] (overall ratio of fluorescence intensity of WT to PTAP^–^YP^–^ mutant approximately equal to 7×) and many cells infected with the PTAP^–^YP^–^ lacked p17 fluorescence (Additional file 4: Figure S4C). This supports the notion that the PTAP^–^YP^–^ virus buds are arrested prior to bud scission, and consequently also to proteolytic maturation. In these MDMs the immunostained virus puncta were also located primarily in IPMCs. For cells where the 3D confocal stacks indicated virus staining both in the IPMC and at the cell surface (and where IPMCs were clearly separated from the cell surface), the IPMC staining was segmented and measured relative to the total p24/p55 fluorescence of the cell. This demonstrated that, on a per cell basis, more than 90 % of the virus labelling was in IPMCs (Fig. 3g). Thus, for MDMs that express IPMC domains, the IPMC is the main site where HIV assembles. The higher levels of cell surface viruses on cells infected with HIV-1 WT is consistent with higher levels of infection and larger numbers of viruses, and suggests that some HIV particles may be re-directed to the PM as the IPMC becomes crowded.

These studies strongly support the view that HIV is predominantly assembled at IPMCs in primary human macrophages.

### Ultra-structural analysis of HIV assembly sites in infected primary MDMs

**Fig. 4:**
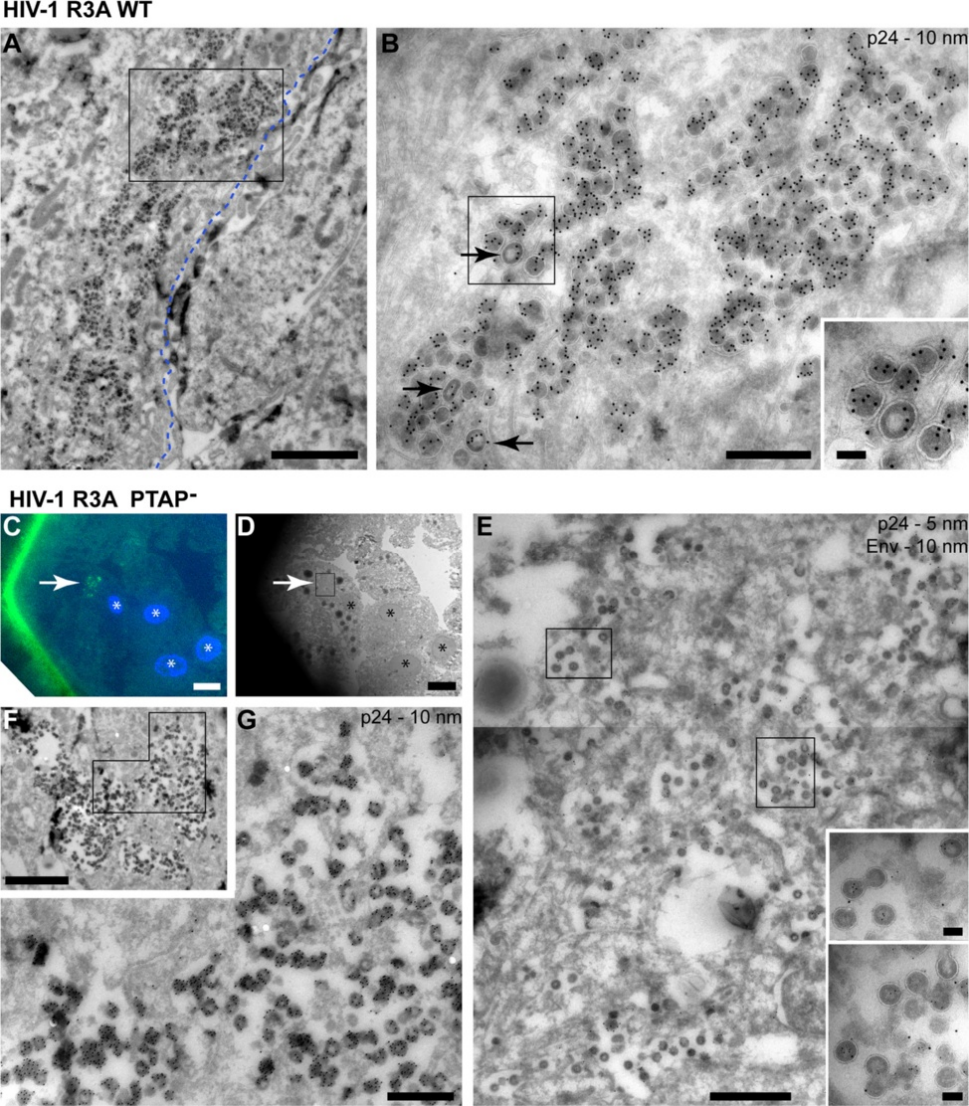
Electron microscopy (EM) immunolabelling of cryosections from monocyte-derived macrophages (MDMs) infected with HIV-1 R3A WT or the PTAP^−^ mutant. Seven-day-old MDMs were infected with HIV-1 R3A WT or the rescued release-defective HIV-1 R3A PTAP^−^. After a further 7 days, cells were fixed and processed for cryosection immunolabelling. Ultrathin cryosections were immunolabelled with antibodies against p24/55, a fluorescent rabbit-anti-mouse bridging antibody and protein A-gold, or double-labelled for Env as indicated. **a, b** MDMs infected with HIV-1 R3A WT. Many cells contained intracellular plasma membrane-connected compartments (IPMCs) packed with electron-dense mature virus profiles. The approximate location of the cell surface is indicated by the *dashed blue line*.* Arrows* indicate occasional immature viruses (detail shown in the inset). **c–g** MDMs infected with the HIV-1 R3A PTAP^−^ mutant. **c** On-grid immunofluorescence staining with the fluorescent bridging antibody (*green*) shows the location of a cell with IPMC near the corner of grid bars (*white arrow*). **d** EM image of the same cell. Note the characteristic pattern of the four nuclei (marked with *) for orientation, indicating the location of the IPMC (*white arrow*). **e** A higher power montage image of the boxed area in (**d**) showing the intracellular membranes with associated immature virus buds (see insets). **f** A similar view of an IPMC from another experiment. The marked area is shown enlarged in (**g**). Scale bars: **a**, 2 μm; **b**, 500 nm; **c**, **d**, 10 μm; **e**, 1 μm; **f**, 2 μm; **g**, 500 nm; and all insets, 100 nm

To analyse the exact locations where the viruses assemble, MDM cultures infected with HIV-1 R3A WT or the rescued PTAP^–^ or PTAP^–^YP^–^ proviruses were fixed and processed for cryosectioning and immuno-EM, and ultrathin sections were stained with antibodies against the HIV Gag protein and protein A-gold. Examination of MDMs infected with R3A WT showed frequent infected cells with extensive IPMCs packed with gold-labelled viruses (Fig. 4a, b). At high magnification, the majority of the particles had electron-dense cores, and sometimes the triangular or circular profiles of the truncated cone-shaped cores of mature virus particles could be distinguished. Occasional particles also showed the characteristic ring of intact p55Gag indicative of immature virus particles (Fig. 4b and inset).

**Fig. 5:**
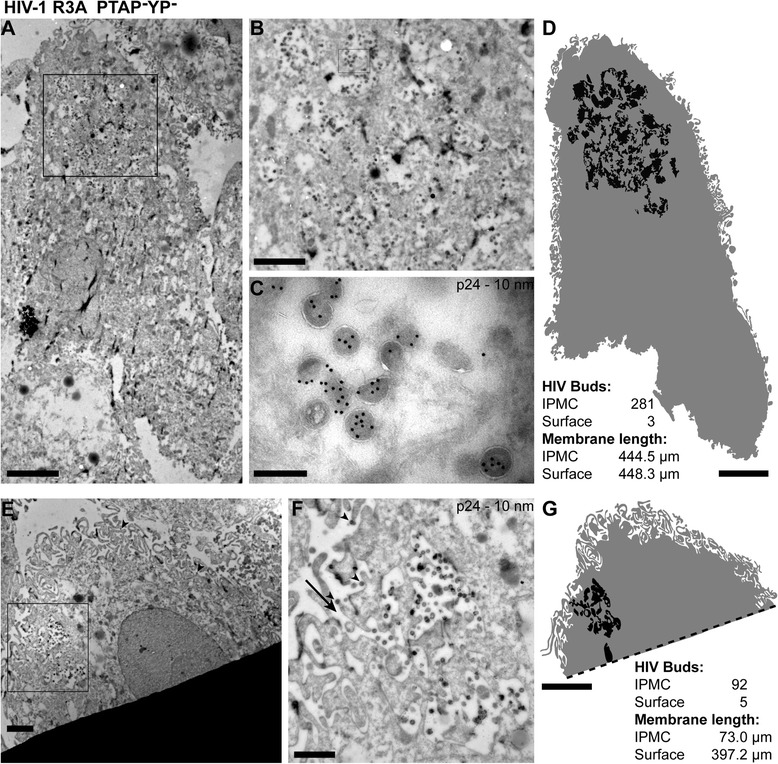
Immuno-electron microscopy (EM) of monocyte-derived macrophages (MDMs) infected with HIV-1 R3A PTAP^−^YP^−^. Seven-day-old MDMs were infected with HIV-1 R3A PTAP^−^YP^−^ for a further 7 days, fixed and processed for cryosection immunolabelling with antibodies against p24/55, and protein A-gold as in Fig. 4. A rabbit-anti-mouse Alexa-488 bridging antibody was used to locate infected cells by on-grid correlative light and EM. **a** A representative cell from Donor 429 located by on-grid immunolabelling. The marked area from the intracellular plasma membrane-connected compartment (IPMC) is shown at higher magnification in (**b**). **c** View of the area marked in (**b**) to show the immature phenotype of the budding-arrested virus particles. **d** Segmentation of the cell surface (*grey*) and IPMC membranes (*black*). Virus buds were counted and the lengths of membrane in the section calculated from the segmentations. **e**–**g** A partial cell profile from another infected cell, Donor 432. **e** Low power overview of the cell profile as seen emerging from under the grid bar. **f** Higher magnification detail of the marked area showing the IPMC. The *arrow* indicates an opening from the IPMC to the cell surface and *arrowheads* in (**e**) and (**f**) mark the five immature virus particles at the cell surface. **g** Segmentation of this cell as in (**d**). The length of the grid bar indicated by the dashed line was not included in the analysis. Scale bars: **a**, **d** and **g**, 5 μm; **b** and **e**, 2 μm; **c**, 200 nm; and **f**, 1 μm

The number of infected cells was lower for cultures infected with the PTAP^–^ or PTAP^–^YP^–^ preparations, probably because budding arrested mutants, in contrast to HIV-1 WT viruses, are unable to establish spreading infections. For the mutants we therefore used correlative on-grid immunofluorescence staining to locate infected cell profiles. Ultrathin cryosections were placed on finder grids and labelled with anti-p24/p55 antibodies, a fluorescent rabbit anti-mouse bridging antibody and protein A-gold. Fluorescence imaging was used to identify infected MDMs and re-find the cells in the EM (Fig. 4c, d). At higher power, the fluorescent staining correlated with IPMC structures filled with gold-labelled immature virus profiles (Fig. 4e–g). Notably, most cell profiles lacked arrested buds directly at the cell surface. Similar analysis of MDMs infected with a rescued PTAP^–^YP^–^ virus stock is shown in Fig. 5. Again, the IPMCs usually consisted of complex membrane mesh-works with interconnected pockets and associated immature virus buds. To better understand the structures of the compartments, cell profiles with labelled virus buds were photographed at high magnification and the cell surface and membranes lining the virus-containing IPMCs were traced. This revealed the very complex nature of the compartments (Fig. 5d, g). In many of the cell profiles, IPMCs were found deep within the cell, while other cell profiles showed IPMC structures close to or joining with the cell surface. Since EM allowed us to identify individual budding figures, we counted virus profiles within IPMCs or directly at the cell surface, and the 2D lengths of membrane in the sections were calculated from the tracings. Overall, we analysed cell profiles from four blood donors infected with either PTAP^–^ or PTAP^–^YP^–^ viruses (Table 1 and Additional file 5: Table S1). More than 95 % of all the HIV budding profiles were located within IPMC structures. For the density of virus buds per unit membrane, budding profiles in IPMCs were found to be enriched at least 40×, and overall by 57×, compared to the cell surface, though this varied between donors and the different cells examined (Table 1 and Additional file 5: Table S1). These results strengthen the argument that HIV assembly is targeted with high fidelity to the IPMCs.

**Table 1:** Quantitation of virus buds and membrane lengths for cryosections of MDMs infected with budding-arrested HIV-1 mutants

Mutant	No. of cell profiles analysed^a^	Cell surface			IPMC			% of buds in IPMC	Enrichment of buds in IPMC^b^
		No. of virus buds	μm of membrane	Buds/μm of membrane	No. of virus buds	μm of membrane	Buds/μm of membrane		
PTAP^–^									
D415^c^	7 (5)	26	2620	0.0099	1137	1378	0.8248	97.8	83 ×
PTAP^–^YP^–^									
D428	9 (5)	4	2041	0.0020	719	931	0.7723	99.4	394 ×
D429	12 (11)	89	5278	0.0169	1149	1814	0.6333	92.8	38 ×
D432	6 (5)	44	2486	0.0177	417	483	0.8633	90.5	49 ×
Overall total	34 (26)	163	12,424	0.0131	3422	4607	0.7428	95.5	57 ×

Monocyte-derived macrophages (MDMs) from four different donors were infected with the HIV-1 R3A mutants PTAP^–^ or PTAP^–^YP^–^, and infected MDMs located by on-grid immunolabelling. Electron microscopy images were recorded and analysed by counting the number of virus buds seen either at the cell surface or within intracellular plasma membrane-connected compartment (IPMCs). Linear lengths of surface or IPMC membrane within the sections were measured by tracing (see Methods)

^a^ The number of cell profiles for which viral buds were counted and membrane lengths measured (the number of distinct cells analysed is given in brackets)

^b^ The ratio of the density of buds/μm of membrane in the IPMC compared to the cell surface is shown as fold enrichment

^c^ Donor ID

### Imaging HIV assembly sites by serial block-face EM and 3D reconstruction

**Fig. 6:**
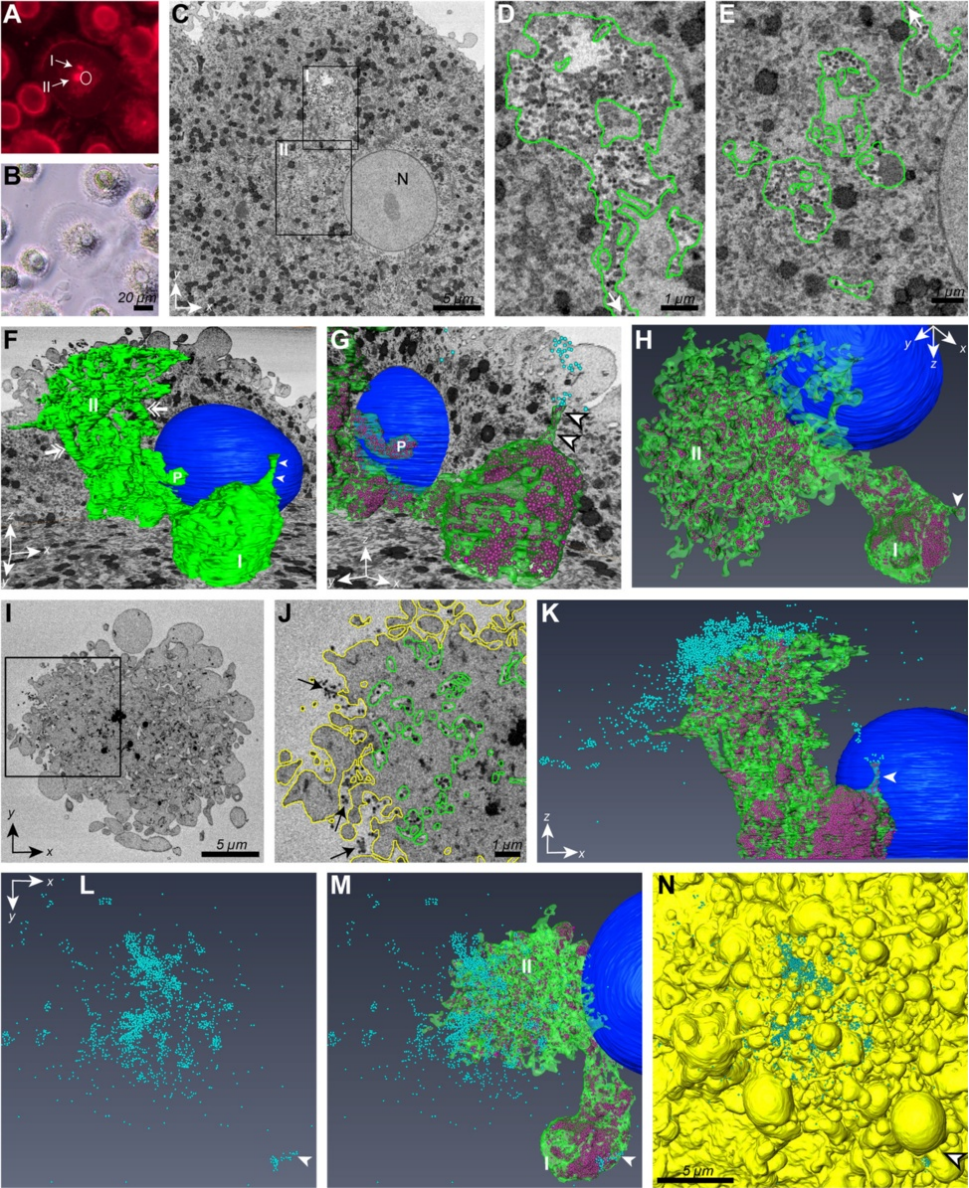
Imaging the intracellular plasma membrane-connected compartment (IPMC) by serial block-face scanning electron microscopy (EM) and 3D reconstruction. Monocyte-derived macrophages (MDMs) were infected with HIV-1 R3A PTAP^−^YP^−^ as in Fig. 5, stained with CellMask orange to visualise the plasma membrane and IPMCs, fixed and processed for serial block-face scanning EM. **a**, **b** Fluorescence and phase contrast images of a selected cell with prominent intracellular staining. The circle in **a** marks the position of the nucleus (based on the phase image) and I and II identify two portions of the IPMC. **c**–**e** Serial block-face EM images from this cell. **c** shows the first of a set of 300 sections. N, nucleus. **d**, **e** Higher magnification details of the two marked areas in (**c**) corresponding to portions I and II of the IPMC, respectively, reveal the presence of virus buds. Membrane boundaries of the compartment were segmented (*green*). The *arrows* mark the connection between the two portions of the IPMC. **f** 3D reconstruction of the IPMC shown with xy and xz ortho-slices to indicate the context of the cell volume. The portions of the IPMC are labelled I and II. Arrowheads mark a short channel connecting portion I to the cell surface, the double-headed arrows show branches associated with region II, and P indicates a pocket from region II. **g** Detail of area I with xy and yz ortho-slices. Internal virus buds are shown in *magenta*. **h** View of IPMC portion II showing the mesh-like interconnected IPMC membranes. **i**–**n** Analysis of virus buds at the cell surface. **i** Detail from section 220. The boxed area is enlarged in (**j**), with the IPMC and cell surface segmented in *green* and *yellow*, respectively. *Arrows* mark clusters of virus buds at the cell surface between membrane protrusions. **k** Side view of the IPMC (xz) with virus buds in the IPMC in *magenta* and at the cell surface in *cyan*. **l**–**n** Views looking down onto the cell surface (xy orientation) showing cell surface virus buds only (**l**), cell surface virus buds with the IPMC (**m**), or cell surface virus buds and the cell surface in *yellow* (**n**). For all reconstructions, the IPMC is shown in *green*, cell surface *yellow*, IPMC virus buds *magenta*, cell surface virus buds *cyan* and the nucleus in *blue*. *White arrowheads* mark the channel from IPMC portion I towards the cell surface (in **f**, **g** and **k**) or the cell surface virus buds above the channel opening in **l**–**n**. Scale bars **b**, 20 μm, **c**, **i** and **n**, 5 μm and **d**, **e** and **j**, 1 μm. See also Additional file 6: Figure S6, Additional file 7: Movie S1, Additional file 8: Figure S7 and Additional file 9: Movie S2

Our studies by correlative light and EM of cryosections captured various random views through infected cells and revealed a variety of arrangements of the IPMCs relative to the cell surface. To determine how these structural views might relate to each other in 3D we used serial block-face EM [37]. MDMs infected with the R3A PTAP^–^YP^–^ mutant were labelled with CellMask orange plasma membrane stain, which can access IPMCs through their surface connections [9], and cells with prominent IPMCs were identified. Herein, we show one cell with a prominent IPMC (Fig. 6a, b). Initial en face ultrathin sections from the basal surface of the cell, adjacent to the coverslip, confirmed that it was infected, with accumulations of viruses in the IPMC. This cell was therefore imaged by serial block-face scanning EM, where we collected a data set of 300 sections, representing a total volume of 32.8 × 32.8 × 15 μm with voxel size 4 × 4 × 50 nm (Additional file 6: Figure S5 and Additional file 7: Movie S1). Selected sections and 3D reconstructions from this cell are shown in Fig. 6 and Additional file 8: Figure S6 and Additional file 9: Movie S2. The first section of the set (section 0) shows the location of the IPMC adjacent to the nucleus, with virus buds visible within the compartment (Fig. 6c–e, see also Additional file 8: Figure S6). Manual segmentation of the IPMC and cell surface membranes allowed us to generate a 3D model of the cell, while virus buds were identified and manually placed in the model using the TrakEM2 module of ImageJ. Various views of the IPMC are shown in Fig. 6f–h. As already apparent from the CellMask stained image, this IPMC consisted of two connected portions (marked I and II in Fig. 6). The smaller portion (marked I, Fig. 6f, g) was relatively compact (diameter approximately 4 μm) and contained large numbers of HIV buds. A narrow channel connected this part of the IPMC to the cell surface (Fig. 6f, g). The adjacent larger portion of the IPMC (marked II) consisted of a complex network of convoluted membranes with narrow connections as well as wider channels and branches, becoming increasingly complex towards the top and eventually opening to the cell surface at multiple sites. The cell surface itself appeared rather convoluted, with numerous membrane processes, protrusions and bleb-like structures.

We also analysed the locations of the budding HIV particles in relation to the IPMC and cell surface membranes. Immature virus particles were found throughout the IPMC, including the channels, branching membrane mesh-works and connections towards the cell surface, indicating that all parts of the IPMC have the potential to support HIV budding. Although most of the cell surface was devoid of viral budding profiles, some buds were noted at the cell surface in higher sections near the top of the cell (Fig. 6i and Additional files 6, 7, 8 and 9). Altogether, we identified 15,264 virus particles in the IPMC and 2085 particles at the cell surface and among the membrane protrusions and blebs, with surface virus buds representing about 12 % of the total viruses in the images from this cell. Notably, these virus buds were not distributed evenly over the cell surface, but appeared concentrated around sites where the IPMC joins the cell surface (Fig. 6k–m and Additional file 8: Figure S6 and Additional file 9: Movie S2). The arrangement of virus buds seen in this cell corresponds to the various different arrangements of buds in IPMCs or at the cell surface seen on the cryosections (see above), where we observed cell profiles with IPMCs deep in the juxta-nuclear area, or close to and connecting with the cell surface.

Notably, since this analysis was done on MDMs infected with mutant HIV unable to complete budding, virtually all assembly events should have been captured for analysis. The predominant localization of HIV buds in IPMCs, seen by immunofluorescence and confocal microscopy, as well as different EM methods, suggests that HIV assembly is specifically targeted to the IPMCs in MDMs.

## Discussion

In this study, we have addressed the question of whether HIV assembly in MDMs preferentially takes place in the IPMCs, or whether the virus also assembles directly at the cell surface and is immediately released into the medium. Using HIV mutants unable to complete the ESCRT-dependent scission reaction, we arrested viruses at their budding sites, which allowed us to capture essentially all HIV assembly events. Analysis of infected cells by confocal microscopy, or of ultrathin cryosections by transmission EM, showed fewer than 5 % of HIV buds at the cell surface and revealed a large enrichment of immature viruses in the IPMCs. Similarly, reconstruction of an infected macrophage by serial block-face scanning EM revealed the intricate structure of the virus-filled IPMC, with cell surface virus buds located predominantly near connections between the IPMC and the cell surface.

The ability to arrest virus budding by mutating the ESCRT-interacting motifs in the p6 portion of Gag confirms that, as in other systems, the ESCRT machinery is also essential for HIV budding in MDMs [8, 11, 29, 38]. For the PTAP^–^ and PTAP^–^YP^–^ mutants, we only detected immature budding profiles. This was clear from staining with an antibody to p17 that only detects the cleaved MA protein. The HIV protease is only active as a dimer, and Gag proteolysis is generally believed to occur concomitant with or shortly after bud scission when the GagPol precursors are able to interact and initiate proteolysis [39]. Although we generally saw little MA staining in cells expressing the budding-arrested HIV mutants, we did notice occasional cleavage in arrested buds, particularly in cells expressing very high levels of Gag. Overall, in MDMs infected with the PTAP^–^YP^–^ mutant, levels of the processed MA were reduced more than 7-fold (Additional file 4: Figure S4C); furthermore, at the EM level, we never detected particles with clear virus cores.

To identify in detail the sites of HIV assembly, large numbers of MDMs isolated from eight different blood donors were analysed by immunofluorescence confocal microscopy and more than 1000 cells were scored for virus distributions (Figs. 2 and 3). Importantly, immunofluorescence staining for Gag p24/55 revealed accumulations of viruses in IPMCs both in MDMs transfected with HIV proviruses and fixed after 24 h (Fig. 2), as well as in cells infected with WT or rescued ESCRT mutant virus preparations and examined 7 days post-infection (Fig. 3). This indicates that virus targeting to the IPMCs is not dependent on how long the cells have been infected. Furthermore, ultrathin cryosections of infected MDMs from four of these donors were studied by EM (Figs. 4 and 5, and Table 1). HIV buds were seen almost exclusively in the IPMCs, indicating that, in MDMs where IPMCs were present, HIV assembly is directed to these compartments. Although all these studies were on fixed cells, live cell imaging studies with Gag-GFP also suggest that Gag assembly is first detected in the IPMCs [8, 9]. Together, these studies show that the accumulations of intracellular HIV particles in MDMs are due to direct targeting of virus budding to the IPMCs and not to extensive internalisation of virus particles.

It is not clear how the virus targets IPMC membranes in preference to the cell surface. Gag binding to the cytoplasmic face of the plasma membrane requires PtdIns(4,5)P_2_, but since PtdIns(4,5)P_2_ is also present at the cell surface [9], there must be other distinguishing features between the membranes of IPMCs and the cell surface. It is possible that there may be variation in the local PtdIns(4,5)P_2_ concentration, or differences in the PtdIns(4,5)P_2_ fatty acid chains that may contribute to local heterogeneity in membrane fluidity or lipid phases (see [40]), allowing stronger Gag binding to IPMCs. Most cellular proteins that have been identified in the IPMCs are also present at the cell surface [4, 7, 10, 11], though again there may be differences in the local abundance of these proteins.

Since it became clear that the virus-containing compartments in MDMs are actually connected to the cell surface [4, 10], there have been studies to delineate the fine structure of these compartments at the EM level. For analysis of the 3D structure of the IPMCs, imaging approaches have included ion-abrasion scanning EM [41], EM tomography [6] or serial section transmission EM [42], which all revealed the complex organisation of the IPMCs with many internal membranes and convoluted interconnected membrane mesh-works. Our serial block-face scanning EM imaging provides a significantly more comprehensive view of IPMCs. We have been able to image a much larger volume than was achieved in previous studies (>50,000 μm^3^), at a resolution comparable to EM tomography. Our studies revealed the major portion of a large IPMC in which we were able to identify and count individual immature virus buds. The overall structure of the compartment resembles IPMCs revealed by fluorescence staining with various membrane dyes or the PtdIns(4,5)P_2_-binding pleckstrin homology domain of phospholipase Cδ [9], but the method also revealed the finer channels and connections, especially in the upper mesh-like portion of the IPMC (marked as Portion II in Fig. 6). We have also been able to define the surface connections where IPMCs open to the exterior of the cell, including a single channel from one part of an IPMC opening in the deep fold between bleb-like cell surface protrusions, or a very complex branching and anastomosing membrane web merging with the upper surface of a cell.

Overall, we have been able to identify more than 15,000 immature virus buds in this IPMC, associated with all parts of the structure, although the densities of the buds varied in different parts of the IPMC. Since the very basal portion of the IPMC is missing from our model, this cell would have even more intracellular virus particles. We also analysed viruses at the cell surface and found that they were highly concentrated above the areas where the IPMC opens to the cell surface. Many particles were seen in groups and clusters, sometimes attached to remaining membrane extensions. This was true for the channel connection (Arrowheads in Fig. 6 – note the two small groups of viruses above the channel opening, but as the channel opening is hidden deep between large membrane blebs, only the upper cluster of virus buds is visible from the cell surface) as well as for the larger accumulations of surface virus buds over the centre of the cell above the larger part of the IPMC. Normally, these viruses would be released into the medium and not be observed, but here the use of budding-arrested mutants has allowed us to define in detail the sites where viruses are able to exit the IPMCs.

Although we have only been able to analyse one cell at this resolution, the structure of the IPMC in this cell reflects arrangements of IPMC structures and viral budding sites seen for the MDM profiles analysed for the cryosections (Figs. 4, 5 and Additional file 5: Table S1), where we were able to examine multiple cells from several donors. Here, we observed cell profiles with deep IPMCs removed from the cell surface (e.g. Fig. 4e, f) or others close to and connecting to the cell surface (e.g. Fig. 5e–g) and cell profiles with channel-like connections to the cell surface (not shown). Notably, we even detected two cell profiles that lacked IPMCs (in the sectioned area), but with immature arrested HIV buds among cell surface membrane sheets and microvilli (Additional file 5: Table S1: D429 cell profiles 18 and 26, with virus only at the cell surface) revealing the sensitivity of the correlative EM analysis and demonstrating that we were not biased towards cell profiles with viruses in IPMCs for this analysis. In addition, the cryosection dataset included a number of cells with prominent cell surface blebs. Together with other published information, the cell shown in Fig. 6 appears to be representative of a typical HIV-infected macrophage in culture.

Macrophages play key roles in HIV infection. Tissue macrophages are found in all parts of the body, and are able to transfer viruses to T cells via virological synapses. As infected macrophages are relatively long-lived, they are able to store and archive infectious virus particles, and they may be particularly important in disseminating viruses late in HIV infection in the brain. The present study reveals the detailed structure of the IPMC and demonstrates that HIV is specifically targeted to this compartment. Understanding the structure of the IPMCs may also shed light on the role of HIV-infected macrophages as a virus reservoir – the assembled virus particles appear sequestered within the IPMC environment, largely protected from the immune response [42], but able to infect CD4^+^ T cells following regulated release of HIV via virological synapses [17, 18]. In this way, macrophages and their specialised IPMCs directly contribute to HIV pathogenesis.

## Conclusion

To investigate the fidelity of HIV-1 targeting to IPMCs in human MDMs, we used mutants unable to complete the ESCRT-dependent scission reaction to arrest viruses at their budding sites, allowing us to capture all HIV assembly events. Detailed morphological analysis of the distribution of the budding-arrested viruses by immunofluorescence staining and confocal microscopy, as well as by EM, showed fewer than 5 % of the HIV buds at the cell surface, thereby demonstrating specific targeting of HIV assembly to IPMCs. High resolution reconstruction and segmentation of an infected macrophage by serial block-face scanning EM revealed the detailed ultrastructure of an IPMC within a large cell volume, and allowed us to observe the location of individual budding events. These studies demonstrate that HIV assembly is specifically targeted to complex IPMCs and indicates HIV particles stored in these protected sites may function as virus reservoirs.

## Methods

### Reagents and antibodies

Unless indicated otherwise, tissue culture media and supplements were obtained from Life Technologies (Paisley, UK), tissue culture plastic from Thermo Fisher Scientific (Cramlington, UK) or Techno Plastic Products AG (Trasadingen, Switzerland) and chemicals were obtained from Sigma-Aldrich (Dorset, UK). Restriction enzymes and the DNA ladder were purchased from Promega (Southampton, UK) and EM chemicals and supplies from Agar Scientific (Stansted, UK) or TAAB Laboratories (Reading, UK).

Antibodies against HIV-1 p24/p55 (38:96 K and EF7, ARP365 and ARP366, respectively), p17 (4C9, ARP342) or Env (2G12, ARP3064) were obtained from the National Institute for Biological Standards and Control, Center for AIDS Reagents (NIBSC CFAR, South Mimms, UK; http://www.nibsc.org/science_and_research/virology/centre_for_aids_reagents.aspx). The mouse monoclonal antibody Kal-1 against HIV-1 p24 was from Dako (Thetford, UK; Dako Cat# M0857, RRID:AB_2335686). The rabbit polyclonal anti-ALIX antibody was a gift from Wes Sundquist (University of Utah, Salt Lake City, USA). Anti-CD44 (MEM-85; Abcam Cat# ab2212 RRID:AB_302891) and anti-VDAC-1 (ab15895; Abcam Cat# ab15895 RRID:AB_2214787) were from Abcam Ltd. (Cambridge, UK), anti-Tsg101 (M-19) from Santa Cruz Biotechnology (Cat# sc-6037 RRID:AB_2208099), and mouse anti-adaptin-γ (clone 88; BD Bioscience Cat# 610386 RRID:AB_397769) from BD Bioscience. Horseradish peroxidase-conjugated goat anti-mouse and anti-rabbit were purchased from Life Technologies (Thermo Fisher Scientific #32430 RRID:AB_1185566; #31460 RRID:AB_228341, respectively), horseradish peroxidase-conjugated donkey anti-goat from Santa Cruz (Santa Cruz #sc-2020 RRID:AB_631728) and the Alexa Fluor-labelled secondary antibody reagents were from Life Technologies (Thermo Fisher Scientific #A21121 RRID:AB_2535764; #A21134 RRID:AB_10393343; #A21242 RRID:AB_2535811; #A21135 RRID:AB_2535774; #A11037 RRID:AB_2534095 and A11059 RRID:AB_2534106).

### Cell lines and macrophage preparation

HEK 293 T and TZM-bl cells were maintained in Dulbecco’s Modified Eagle’s Medium (DMEM) supplemented with 10 % v/v fetal bovine serum (FBS), 2 mM L-glutamine, 100 units/mL penicillin and 100 μg/mL streptomycin. MDMs were prepared from peripheral blood mononuclear cells isolated from buffy coats obtained from healthy blood donors (National Blood Services, Essex, UK) as described previously [7, 18]. Monocytes were differentiated with macrophage-colony stimulating factor (M-CSF, 10 ng/mL; R&D Systems, Abington, UK) and cultured in Roswell Park Memorial Institute (RPMI) 1640 medium supplemented with 10 % human serum, 100 units/mL penicillin and 100 μg/mL streptomycin.

### Plasmids and viruses

The HIV-1 construct pNL4.3-R3A, a chimeric virus containing the *env* gene of the HIV-1 R3A strain [43] in the NL4.3 backbone, was a gift from J. Hoxie (University of Pennsylvania, Philadelphia, USA). A Gag fragment containing the entire p6 domain was excised from pNL4.3-R3A with EcoRI and ApaI and subcloned into pEGFP-Nl (Addgene). Using the QuickChange® Site-Directed Mutagenesis Kit (Agilent Technologies, Wokingham, UK), the new plasmid carrying the EcoRI/ApaI fragment from pNL4.3-R3A (hereafter, called “carrier plasmid”) was mutagenised at the PTAP and YP residues, or a stop codon was introduced at the beginning of p6 using the following primers (5′ to 3′): R3A_PTAP forward, TTTTCTTCAGAGCAGACCAGAGCTAATACGCCTACCAGAAGAGAGCTTCAGGTTTG; R3A_PTAP reverse, CAAACCTGAAGCTCTCTTCTGGTAGGCGTATTAGCTCTGGTCTGCTCTGAAGAAAA; R3A_YP forward, CGATAGACAAGGAACTGTCTCGTTTAGCTTCCCTCAGATC; R3A _YP reverse, GATCTGAGGGAAGCTAAACGAGACAGTTCCTTGTCTATCG; R3A_p6Del forward, CCCACAAGGGAAGGCCAGGGAATTTTTAACAGAGCAGACCAGA; R3A_p6Del reverse, TCTGGTCTGCTCTGTTAAAAATTCCCTGGCCTTCCCTTGTGGG. The mutagenised carrier fragments were excised from the carrier plasmid and re-inserted into pNL4.3-R3A, generating the mutant proviruses YP^−^, PTAP^−^, PTAP^−^YP^−^ and Δp6. pCMVGag WT was generated from Gag-GFP in pEGFP-N1 (Herminda-Matsumoto and Resh 2000, provided by W. Sundquist) by deleting the GFP sequence. Stocks of infectious HIV-1 R3A PTAP^−^ or PTAP^−^YP^−^ were prepared by transfecting HEK 293 T cells with a mixture of pNL4.3-R3A PTAP^−^ or PTAP^−^YP^−^ and pCMVGag WT. After 24 h, media were collected, the rescued viruses were purified by ultracentrifugation through a 20 % w/v sucrose cushion and stored frozen in RPMI 1640. Virus titres were measured on TZM-bl indicator cells, using the Galacto-Star Reporter Gene Assay System (Life Technologies) as previously described [7, 18].

### Infections and transfections

HEK 293 T cells were transfected with FuGENE HD (Roche, Welwyn Garden City, UK). Fourteen-day-old MDMs were transfected by electroporation using the Amaxa Human Macrophage Nucleofector Kit and Amaxa Nucleofector II (Lonza, Kent, UK), and incubated as described [9]. MDMs were infected with HIV-1 WT or the mutant viruses at 3 and 6 focus forming units per cell (FFU/cell), respectively, by spinoculation (centrifugation at 1300 *g* and 25 °C for 2 h) and cultured for 7 days as described [7].

### Analysis of HIV release

For released viruses, culture supernatants were cleared by centrifugation, overlaid onto 20 % sucrose in PBS and centrifuged at 100,000 *g* for 2 h at 4 °C. Cells were washed in PBS and cells or the purified virus pellets were lysed in Laemmli reducing sample buffer (Sigma-Aldrich, Dorset, UK), heated for 10 min at 95 °C and resolved, blotted and analysed with appropriate antibodies as described before [7, 9, 18]. Signal intensities on western blots were quantified using ImageJ. Virus release efficiencies were calculated as the amount of p24 + p55 in the virus blot relative to the total p24 and p55 in cell and virus lysates.

### Confocal microscopy

MDMs on coverslips were fixed in 4 % formaldehyde, washed, quenched in 50 mM NH_4_Cl in PBS and permeabilized with 0.1 % Triton X-100 in blocking buffer (6 mg/mL purified human IgG and 0.5 % BSA in PBS). Cells were incubated in primary and secondary antibodies as described previously [7, 9]. Nuclei were counter-stained with 0.5 μg/mL Hoechst 33258 (Life Technologies), and the coverslips were mounted with Mowiol (Merck Millipore, Darmstadt, Germany). Confocal images were acquired with a Leica TCS SP3 confocal microscope, 63× oil objective and Leica LAS AF Software (Leica Microsystems, Milton Keynes, UK). Images were processed using ImageJ and assembled using Adobe Illustrator CS4.

### Preparation of cells for cryosectioning and EM immunolabelling

Cells were prepared for cryosectioning as previously described [4, 7]. Briefly, cells were fixed in 4 % formaldehyde in 0.1 M sodium phosphate buffer, pH 7.4, embedded in 12 % gelatine, infiltrated with 2.3 M sucrose and frozen in liquid nitrogen. Samples were screened by immunofluorescence staining of semi-thin cryosections (0.5 μm) as described [7] and imaged with an Axioskop microscope (Carl Zeiss, Hertfordshire, UK) fitted with a charge-coupled device camera (Orca-ER; Hamamatsu), controlled by OPENLAB 5.0.2 software (Improvision, Perkin Elmer). Images were processed using Adobe Photoshop CS4.

EM immunolabelling was as described [4, 44, 45]. Briefly, ultrathin cryosections (~50 nm) on formvar/carbon-coated EM grids or H6 finder grids were quenched in 50 mM glycine/50 mM NH_4_Cl, washed and stained with mouse antibodies to p24/p55 prepared in 2 % BSA, 0.2 % acetylated BSC (BSA-c, Aurion, Wageningen, The Netherlands) in PBS, rabbit anti-mouse Alexa-488 and Protein A-gold (PAG, The EM Lab, Utrecht University, The Netherlands). Some grids were double labelled with antibodies to HIV Env, fixed in 1 % glutaraldehyde, washed, quenched and then stained for p24/p55, as above [7]. For correlative on-grid light and EM, sections were incubated with Hoechst 33258 to stain the nuclei, mounted in 50 % glycerol in H_2_O and fluorescent cells were imaged with an Axioskop microscope (Zeiss) as above. All sections were stained with 2 % neutral uranyl acetate and embedded in 0.4 % uranyl acetate in 2 % methylcellulose [45]. Specimens were examined with a Tecnai 20 (FEI, Eindhoven, The Netherlands) using a Morada camera and iTEM software (Olympus Soft Imaging Solutions), and processed with Adobe Photoshop CS4.

### Conventional EM of Epon-embedded cells

HEK 293 T cells were fixed for 30 min in 1.5 % glutaraldehyde, 4 % formaldehyde in 0.1 M sodium cacodylate buffer, pH 7.4, post-fixed in reduced osmium (1.5 % potassium ferricyanide, 1 % osmium tetroxide) at 4 °C for 1 h, and treated with 1 % tannic acid in 0.05 M sodium cacodylate for 45 min in the dark. The cells were washed with 1 % sodium sulphate in 0.05 M sodium cacodylate, and dehydrated and embedded in Epon 812 resin. Ultrathin sections were stained with lead citrate and imaged with a Tecnai 20 EM as above.

### Serial block-face scanning EM

Seven-day-old MDMs were infected with HIV-1 R3A PTAP^−^YP^−^ for 7 days and incubated in 5 μg/mL CellMask orange plasma membrane stain (Invitrogen Molecular Probes, Paisley, UK) for 10 min at 37 °C. Cells were fixed in 4 % formaldehyde, washed and imaged on an inverted light microscope (DMIL LED; Leica, Vienna, Austria) to identify cells with IPMCs, and bright-field and fluorescence images of the cells of interest were acquired at low magnification. To mark the selected cells, a ring of adjacent cells was removed by scratching manually with forceps. This allowed the region of the coverslip (and later, the resin block) containing the cell of interest to be easily visible to the naked eye, once the cells were osmicated. Cells were then further fixed with 2 % formaldehyde, 1.5 % glutaraldehyde and incubated with reduced osmium at 4 °C. The cells were treated with 1 % thiocarbohydrazide, before a 30 min incubation in 2 % osmium tetroxide and an overnight treatment with 1 % uranyl acetate. A final en bloc stain was performed with lead aspartate before the sample was dehydrated in ethanol and embedded in Durcupan resin.

Embedded EM blocks were examined to identify the cells of interest, trimmed, and ultrathin sections of the cells of interest were taken and examined by transmission EM to confirm virus infection. The region of interest was then excised and mounted with conductive silver epoxy resin onto specimen pins. Samples were further trimmed before being coated with gold palladium and mounted in the 3View microtome (Gatan, CA, USA) coupled to a Sigma VP scanning electron microscope (SEM; Zeiss, Cambridge, UK). Once aligned, the sample and microtome were returned to the SEM chamber and pumped to a vacuum pressure of 5 Pa. The region of interest on the block face was re-located in the SEM using backscattered electron detection and the imaging and cutting parameters were optimised. The voltage was set at 2 kV using the high current setting and a 20 μ aperture, and imaging parameters were set at 8 k × 8 k pixels, with a horizontal field width of 32.8 μm and a pixel size of 4 nm, with a slice thickness of 50 nm. For segmentation, datasets were imported into Amira (FEI VSG, France), and cells of interest were manually segmented, reconstructed and rendered in 3D. The locations of virus buds were manually marked on the sections using the TrakEM2 module of ImageJ, coordinates of the virus particles were calculated using a custom python script [46] and loaded into Amira for visualization.

## Abbreviations

EM, electron microscopy; ESCRT, endosomal sorting complexes required for transport; IPMC, intracellular plasma membrane-connected compartment; MDM, monocyte-derived macrophage; PtdIns(4,5)P_2_, phosphatidylinositol-4,5-bisphosphate

## Additional files

Additional file 1: Figure S1. siRNA-mediated depletion of Tsg101 and/or ALIX has a minimal effect on virus release in monocyte-derived macrophages (MDMs). (**A**) Diagram showing the experimental set-up for siRNA knockdown in MDMs. Seven- or 8-day-old MDMs were infected with HIV-1 BaL (3 FFU/cell), on day 0. One day later, duplicate culture wells per donor were transfected with the appropriate siRNAs (Stealth siRNAs for ALIX, HSS115204; Tsg101, HSS111013; or negative control, 12935-300) using Lipofectamine^TM^ RNAiMAX (Life Technologies) and cultured for 6 days. Media containing virus released during a 24-h window from 6–7 days after infection were collected and concentrated by ultracentrifugation through sucrose cushions. The virus pellets and cells were lysed and analysed by western blotting. Blots for VDAC-1 and adaptin-γ were included as loading controls. (**B, C**) Single knockdown of either ALIX or Tsg101 (**B**), or double knockdown of both proteins (**C**). For analysis, unsaturated band signal intensities were quantified using ImageJ. Knockdown of ALIX or Tsg101 are shown by comparison to the control siRNA. Virus release efficiencies were calculated as the amount of viral p24 + p55 + the p24 in the cell lysates (in macrophages HIV accumulates in IPMCs, and therefore cell lysate p24 represents mature virus that is budded from the host plasma membrane) divided by the total p24 and p55 in cell and virus lysates. Although ALIX and Tsg101 were efficiently depleted, this had minimal effects on HIV release. Data represent independent experiments with MDMs from four and three blood donors in (**B**) and (**C**), respectively. (TIF 1900 kb) Additional file 2: Figure S2. Generation of release-defective HIV-1 R3A proviruses and preparation of infectious virus stocks. (**A**) Schematic representation of the HIV-1 NL4.3-R3A Gag polyprotein. The amino acid sequence of the p6 subdomain in the WT is shown with the Tsg101 and ALIX binding motifs, PTAP and YPLSTSL, respectively, highlighted in green. For the mutants, sequence changes are marked in red, and dashes denote amino acids identical to the WT. The red asterisk indicates the location of the stop codon in ∆p6. (**B**) Determination of the optimal conditions for rescuing release-defective viruses. HEK 293 T cells were transfected with pNL4.3-R3A WT, PTAP^−^ alone, or PTAP^−^ together with increasing concentrations of pCMVGag that expresses WT p55Gag. Cells were incubated for 24 h, and cell lysates and released viruses analysed by western blotting. (**C**) Quantitative analysis of virion-associated p24 in the medium of cells producing HIV-1 R3A PTAP^−^ virus rescued by co-expression of WT p55Gag. (**D**) Virus stocks were prepared from HIV-1 R3A WT, or from the PTAP^−^ mutant using a 12:1 ratio of pNL4.3-R3A PTAP^−^: pCMVGag WT, and infectious titres determined on TZM-bl cells. For (**C**) and **(D**), error bars represent SD for two independent experiments. (TIF 910 kb) Additional file 3: Figure S3. Distribution of HIV-1 R3A WT or PTAP^–^YP^–^ in infected monocyte-derived macrophages (MDMs). Seven-day-old MDMs were infected with HIV-1 R3A WT or the rescued release-defective PTAP^−^YP^−^ for 7 days, fixed, immunolabelled with anti-p24/55 antibodies (Kal-1, green), p17 (4C9, red) and CD44 (magenta) and examined by confocal microscopy. Selected sections from the confocal series for the cells shown in Fig. 3. Numbers indicate the optical slice in the confocal series. (**A**) HIV-1 R3A WT, the cell shown in Fig. 3a. (**B–D**) HIV-1 R3A PTAP^−^YP^−^, showing the cells in Fig. 3b, c and e, respectively. Note that the cell in (**D**) lacks a CD44^+^ intracellular plasma membrane-connected compartment. Scale bars, 20 μm. (TIF 9159 kb) Additional file 4: Figure S4. Distribution of HIV-1 R3A PTAP^–^YP^–^ in monocyte-derived macrophages (MDMs) and quantitation of Gag fluorescence. (**A**) Selected sections from the confocal series for the infected MDM shown in Fig. 3d, HIV-1 R3A PTAP^−^YP^−^ stained p24/55 (Kal-1, green), p17 (4C9, red) and CD44 (magenta). Numbers indicate the slice in the confocal series (numbering from the bottom to the top of the cell). Yellow arrows indicate some of the rare virus puncta at the cell surface. Scale bar, 20 μm. (**B–C**) Quantitation of fluorescence intensities per cell for MDMs infected with HIV-1 R3A WT or the rescued release-defective PTAP^−^YP^−^ for 7 days for cells from two blood donors as indicated. Total staining with Kal-1 anti-p24/55 (**B**) or 4C9 anti-p17 (**C**) was analysed on 3D images using ImageJ. In (**C**) the symbols below the x-axis represent cells for which no p17 fluorescence was recorded. (TIF 7562 kb) Additional file 5: Table S1. Quantitation of virus buds and membrane lengths for cryosections of monocyte-derived macrophages (MDMs) infected with budding-arrested HIV mutants. MDMs from four different donors were infected with the HIV-1 R3A mutants PTAP^−^ or PTAP^−^YP^−^, and infected MDMs located by on-grid immunolabelling. Electron microscopy images were recorded and analysed by counting the number of virus buds seen either at the cell surface or within intracellular plasma membrane-connected compartments (IPMCs). Lengths of surface or IPMC membrane within the sections were measured by tracing (see Methods). Data from all of the cells summarised in Table 1. (XLSX 46 kb) Additional file 6: Figure S5. Imaging a monocyte-derived macrophage (MDM) infected with HIV-1 R3A PTAP^−^YP^−^ by serial block-face scanning electron microscopy (SEM). Selected sections from the serial block face SEM data set. Section numbers from the first section near the bottom of the cell (000) are indicated in the top right of the panels. Images have been segmented for the intracellular plasma membrane-connected compartment (IPMC; green) or cell surface plasma membrane (yellow). I and II show the two portions of the IPMC and the white circle indicates a narrow channel connecting IPMC portion I to the cell surface (white arrow in section 108). Towards the top of the cell, clusters of virus buds are seen between membrane protrusions at the cell surface (e.g. black arrows in section 200 and 240). Scale bar, 5 μm. (TIF 8739 kb) Additional file 7: Movie S1. Imaging a monocyte-derived macrophage infected with HIV-1 R3A PTAP^−^YP^−^ by serial block-face scanning electron microscopy (SEM). Movie showing all 300 sections from the serial block-face SEM data set, from the bottom of the cell towards the top. Note the complex intracellular plasma membrane-connected compartment structure filled with virus particles, and some cell surface virus buds near the top of the cell. (MOV 16087 kb) Additional file 8: Figure S6. Un-cropped images and additional views from the cell shown in Fig. 6. (**A, B**) Slices 000 and 220 in xy orientation, with the intracellular plasma membrane-connected compartment (IPMC) segmented in green and the cell surface plasma membrane in yellow. (**C–E**) xz side view as in Fig. 6k. The positions of section 000 and section 220 are indicated by dashed lines. (**D**) and (**E**) show the same view as in (**C**) with the cell surface (yellow) transparent (**D**) or opaque (**E**). (**F, G**) Uncropped xy view as in Fig. 6l–n, with cell surface virus particles only (cyan, **F**) or with the cell surface (transparent yellow, **G**). (**H–J**) yz side views, representation as in (**C–E**). Note the clustering of cell surface virus particles (cyan) above IPMC exit sites. (TIF 15003 kb) Additional file 9: Movie S2. Model of the cell shown in Fig. 6. The movie shows four rotations of the model with (1) the intracellular plasma membrane-connected compartment (IPMC) only (green). Note branches connecting different parts of the IPMC. (2) Transparent IPMC, revealing internal virus buds (magenta) and (3) cell surface virus buds (cyan). Rotation (4) also shows the cell surface (yellow). Note the clustering of cell surface virus particles (cyan) above IPMC exit sites. (MOV 13075 kb)
