# Supplementary figures and images for: The intracellular plasma membrane-connected compartment in the assembly of HIV-1 in human macrophages
Source: BMC Biol. 2016 Jun 23;14:50. doi: 10.1186/s12915-016-0272-3 (PMC4919869; doi:10.1186/s12915-016-0272-3)

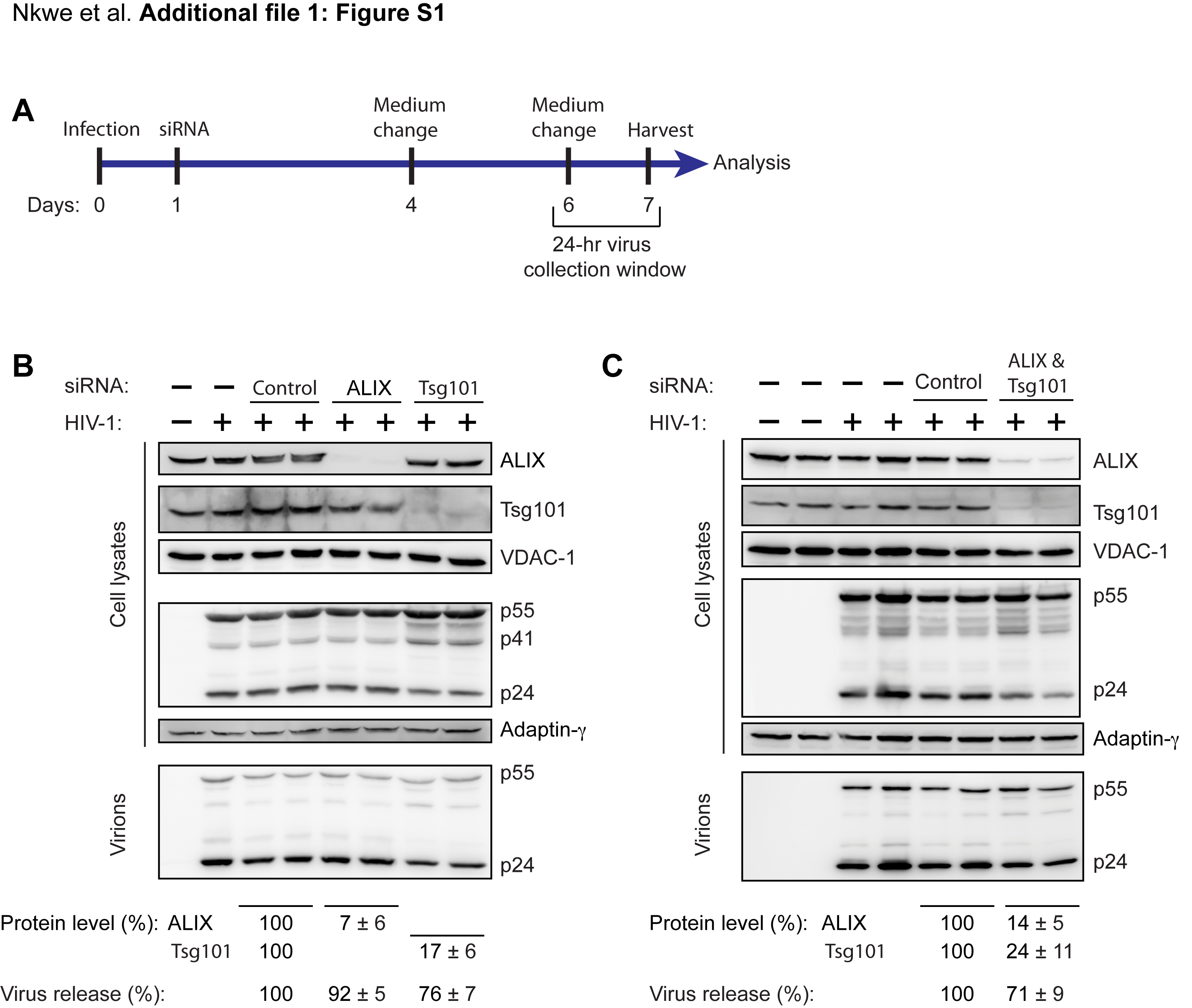

Supplement: Additional file 1: Figure S1. — siRNA-mediated depletion of Tsg101 and/or ALIX has a minimal effect on virus release in monocyte-derived macrophages (MDMs). (A) Diagram showing the experimental set-up for siRNA knockdown in MDMs. Seven- or 8-day-old MDMs were infected with HIV-1 BaL (3 FFU/cell), on day 0. One day later, duplicate culture wells per donor were transfected with the appropriate siRNAs (Stealth siRNAs for ALIX, HSS115204; Tsg101, HSS111013; or negative control, 12935-300) using LipofectamineTM RNAiMAX (Life Technologies) and cultured for 6 days. Media containing virus released during a 24-h window from 6–7 days after infection were collected and concentrated by ultracentrifugation through sucrose cushions. The virus pellets and cells were lysed and analysed by western blotting. Blots for VDAC-1 and adaptin-γ were included as loading controls. (B, C) Single knockdown of either ALIX or Tsg101 (B), or double knockdown of both proteins (C). For analysis, unsaturated band signal intensities were quantified using ImageJ. Knockdown of ALIX or Tsg101 are shown by comparison to the control siRNA. Virus release efficiencies were calculated as the amount of viral p24 + p55 + the p24 in the cell lysates (in macrophages HIV accumulates in IPMCs, and therefore cell lysate p24 represents mature virus that is budded from the host plasma membrane) divided by the total p24 and p55 in cell and virus lysates. Although ALIX and Tsg101 were efficiently depleted, this had minimal effects on HIV release. Data represent independent experiments with MDMs from four and three blood donors in (B) and (C), respectively. (TIF 1900 kb) [file 12915_2016_272_MOESM1_ESM.tif]

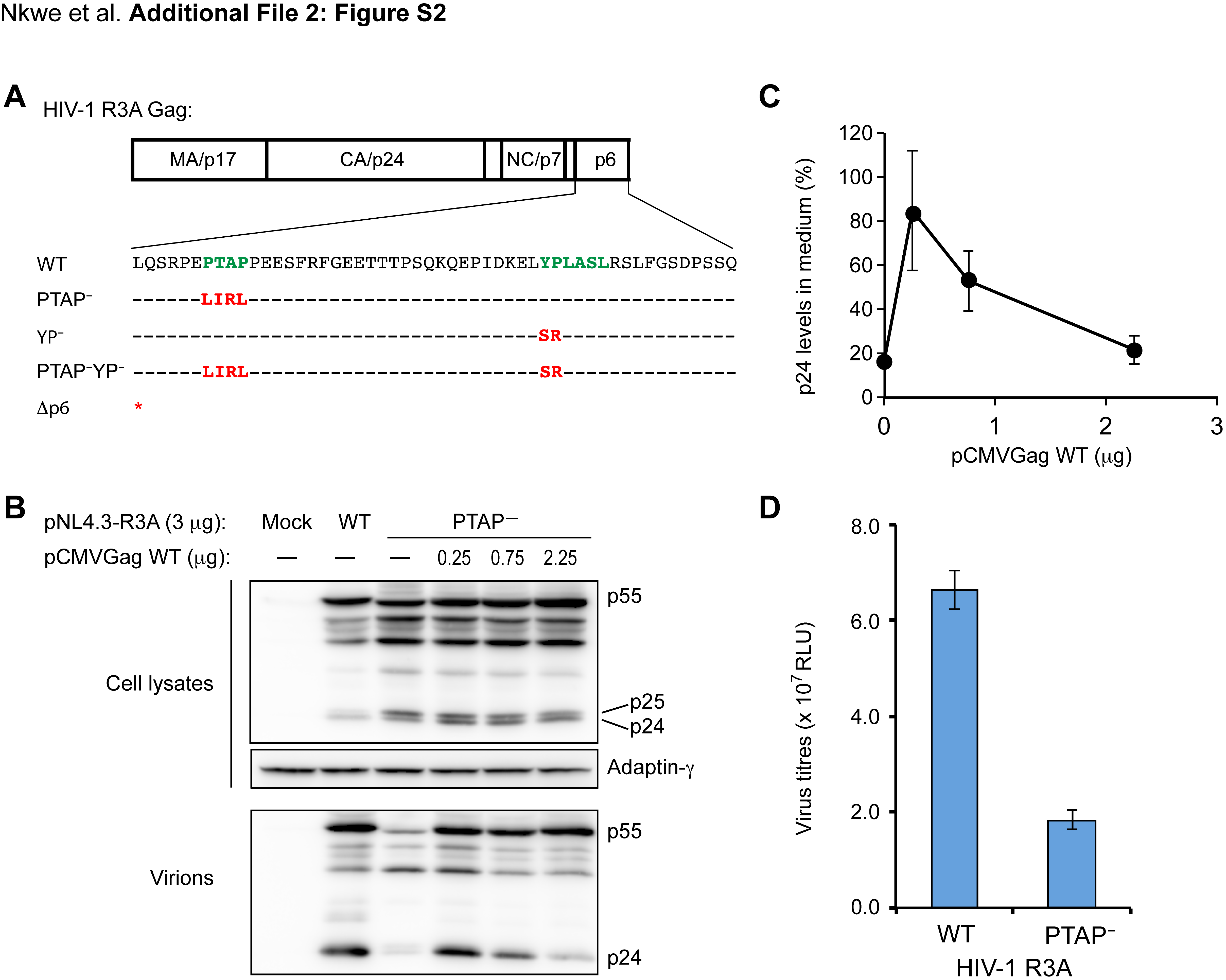

Supplement: Additional file 2: Figure S2. — Generation of release-defective HIV-1 R3A proviruses and preparation of infectious virus stocks. (A) Schematic representation of the HIV-1 NL4.3-R3A Gag polyprotein. The amino acid sequence of the p6 subdomain in the WT is shown with the Tsg101 and ALIX binding motifs, PTAP and YPLSTSL, respectively, highlighted in green. For the mutants, sequence changes are marked in red, and dashes denote amino acids identical to the WT. The red asterisk indicates the location of the stop codon in ∆p6. (B) Determination of the optimal conditions for rescuing release-defective viruses. HEK 293 T cells were transfected with pNL4.3-R3A WT, PTAP− alone, or PTAP− together with increasing concentrations of pCMVGag that expresses WT p55Gag. Cells were incubated for 24 h, and cell lysates and released viruses analysed by western blotting. (C) Quantitative analysis of virion-associated p24 in the medium of cells producing HIV-1 R3A PTAP− virus rescued by co-expression of WT p55Gag. (D) Virus stocks were prepared from HIV-1 R3A WT, or from the PTAP− mutant using a 12:1 ratio of pNL4.3-R3A PTAP−: pCMVGag WT, and infectious titres determined on TZM-bl cells. For (C) and (D), error bars represent SD for two independent experiments. (TIF 910 kb) [file 12915_2016_272_MOESM2_ESM.tif]

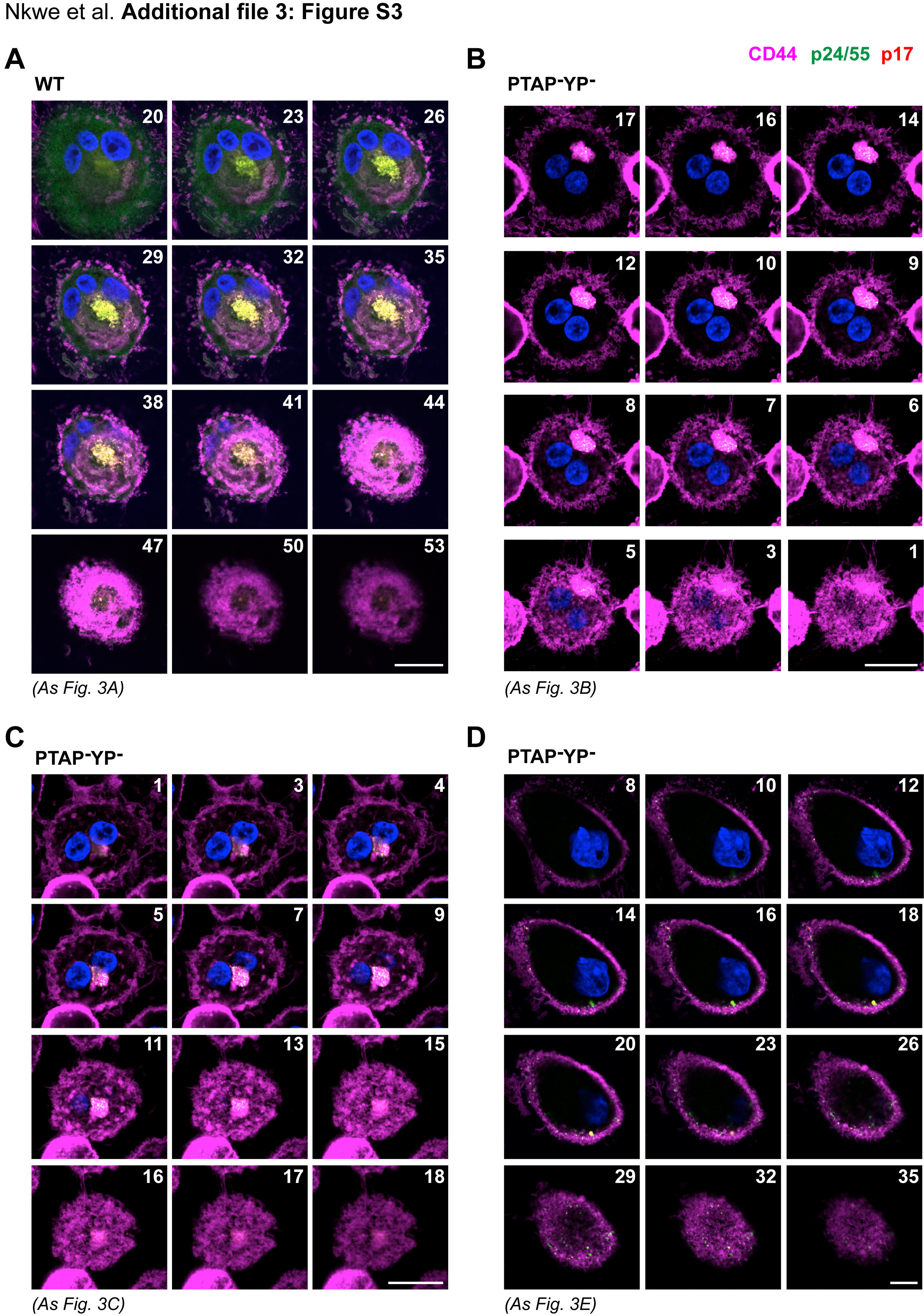

Supplement: Additional file 3: Figure S3. — Distribution of HIV-1 R3A WT or PTAP–YP– in infected monocyte-derived macrophages (MDMs). Seven-day-old MDMs were infected with HIV-1 R3A WT or the rescued release-defective PTAP−YP− for 7 days, fixed, immunolabelled with anti-p24/55 antibodies (Kal-1, green), p17 (4C9, red) and CD44 (magenta) and examined by confocal microscopy. Selected sections from the confocal series for the cells shown in Fig. 3. Numbers indicate the optical slice in the confocal series. (A) HIV-1 R3A WT, the cell shown in Fig. 3a. (B–D) HIV-1 R3A PTAP−YP−, showing the cells in Fig. 3b, c and e, respectively. Note that the cell in (D) lacks a CD44+ intracellular plasma membrane-connected compartment. Scale bars, 20 μm. (TIF 9159 kb) [file 12915_2016_272_MOESM3_ESM.tif]

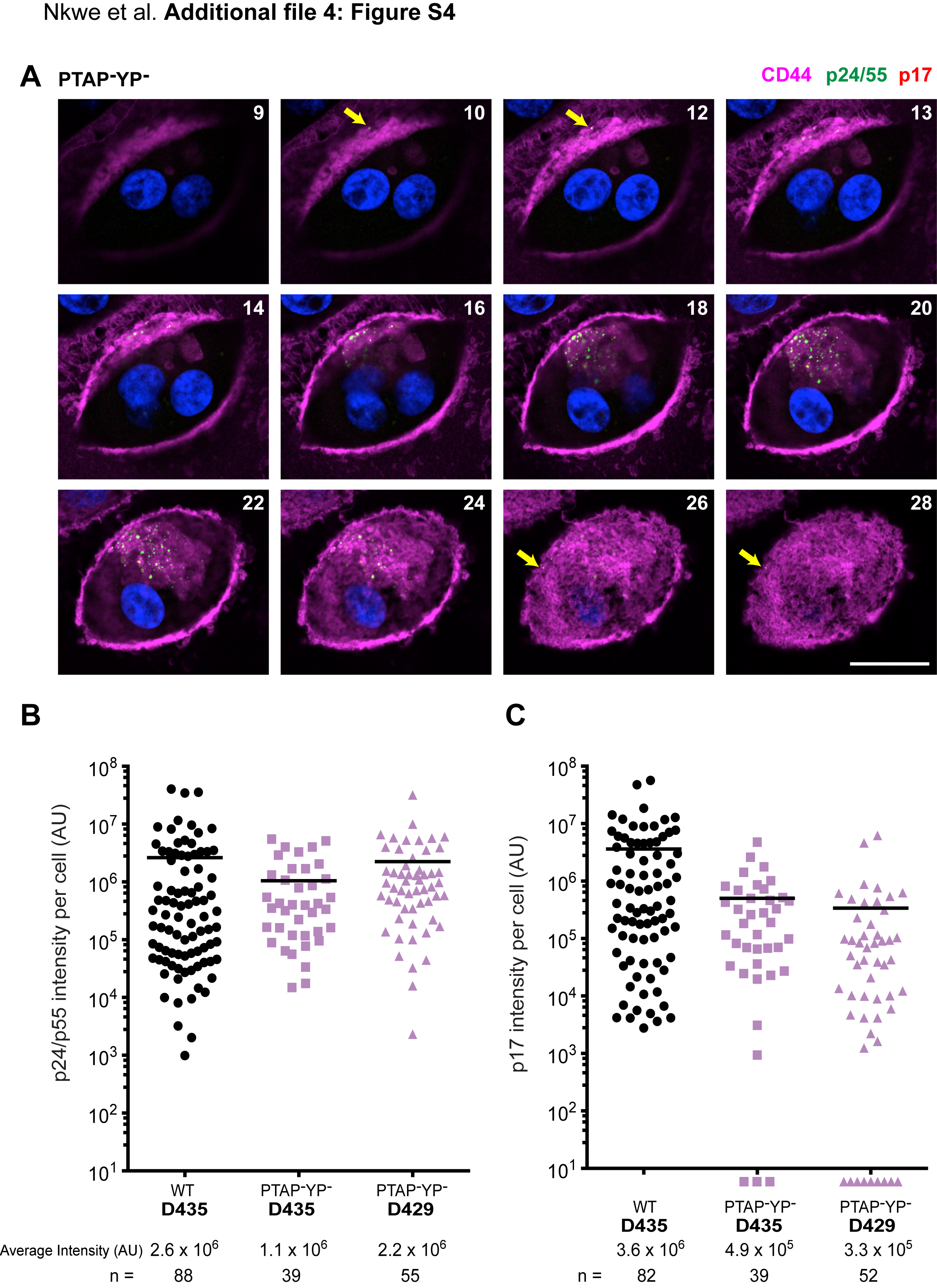

Supplement: Additional file 4: Figure S4. — Distribution of HIV-1 R3A PTAP–YP– in monocyte-derived macrophages (MDMs) and quantitation of Gag fluorescence. (A) Selected sections from the confocal series for the infected MDM shown in Fig. 3d, HIV-1 R3A PTAP−YP− stained p24/55 (Kal-1, green), p17 (4C9, red) and CD44 (magenta). Numbers indicate the slice in the confocal series (numbering from the bottom to the top of the cell). Yellow arrows indicate some of the rare virus puncta at the cell surface. Scale bar, 20 μm. (B–C) Quantitation of fluorescence intensities per cell for MDMs infected with HIV-1 R3A WT or the rescued release-defective PTAP−YP− for 7 days for cells from two blood donors as indicated. Total staining with Kal-1 anti-p24/55 (B) or 4C9 anti-p17 (C) was analysed on 3D images using ImageJ. In (C) the symbols below the x-axis represent cells for which no p17 fluorescence was recorded. (TIF 7562 kb) [file 12915_2016_272_MOESM4_ESM.tif]

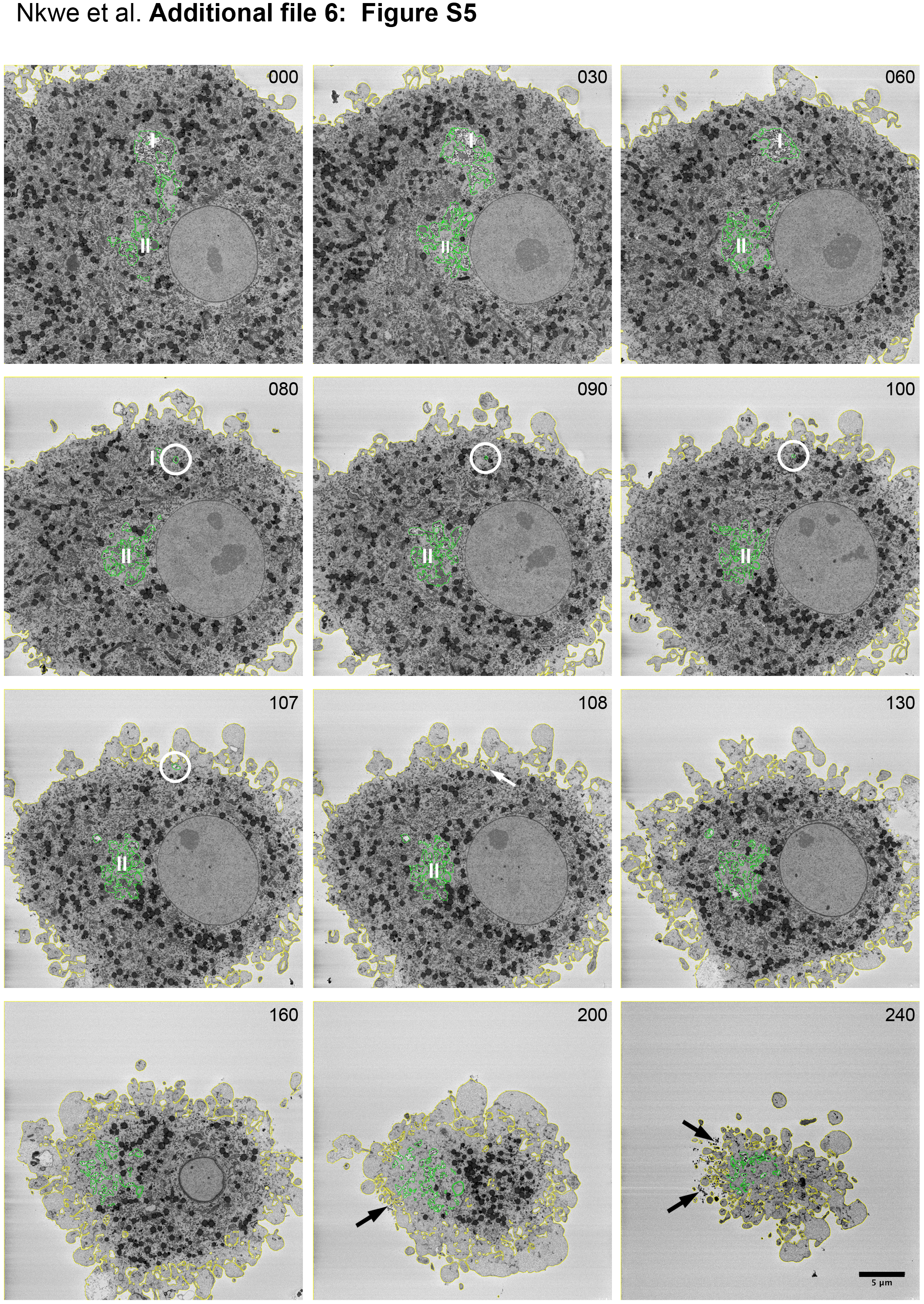

Supplement: Additional file 6: Figure S5. — Imaging a monocyte-derived macrophage (MDM) infected with HIV-1 R3A PTAP−YP− by serial block-face scanning electron microscopy (SEM). Selected sections from the serial block face SEM data set. Section numbers from the first section near the bottom of the cell (000) are indicated in the top right of the panels. Images have been segmented for the intracellular plasma membrane-connected compartment (IPMC; green) or cell surface plasma membrane (yellow). I and II show the two portions of the IPMC and the white circle indicates a narrow channel connecting IPMC portion I to the cell surface (white arrow in section 108). Towards the top of the cell, clusters of virus buds are seen between membrane protrusions at the cell surface (e.g. black arrows in section 200 and 240). Scale bar, 5 μm. (TIF 8739 kb) [file 12915_2016_272_MOESM6_ESM.tif]

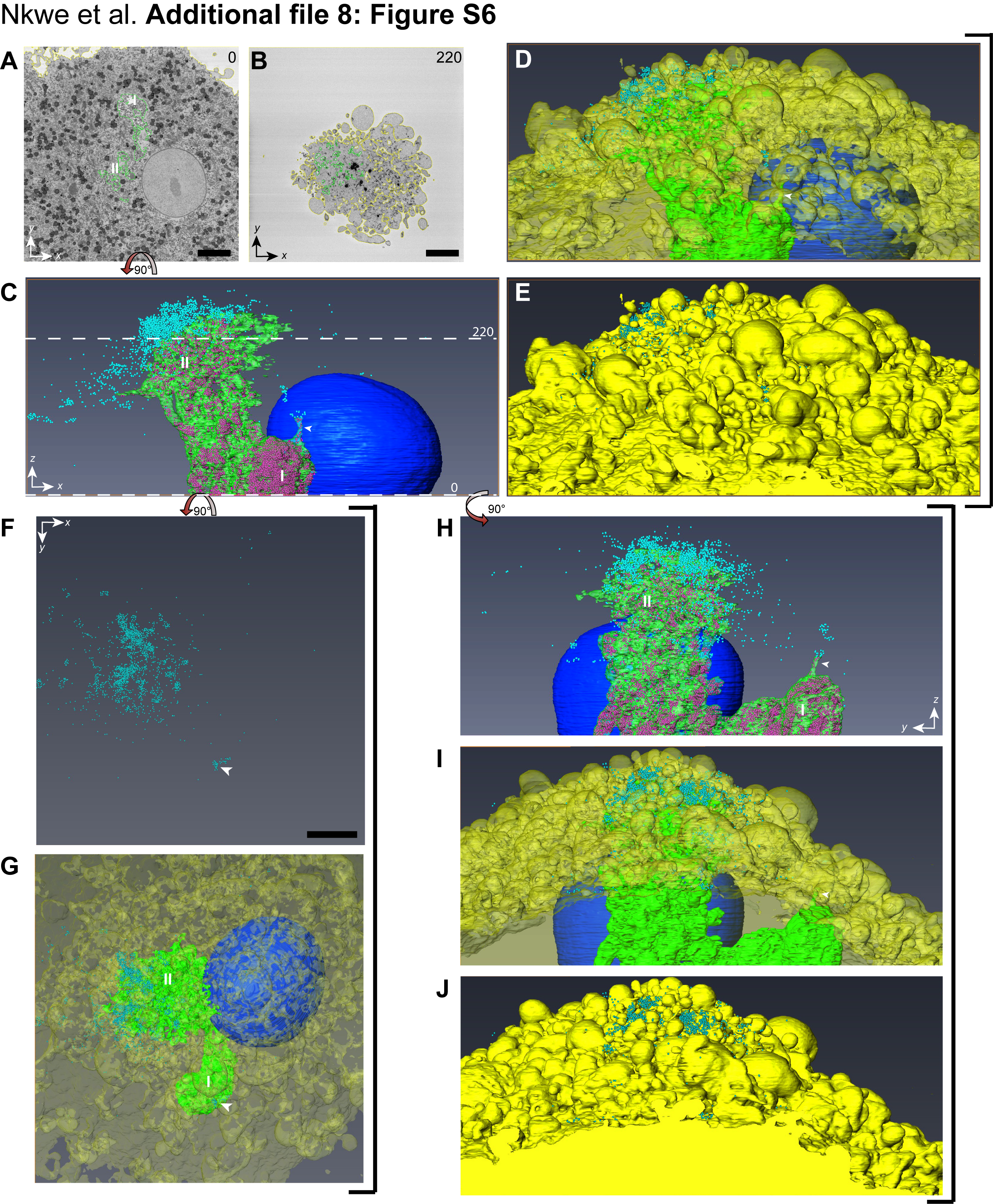

Supplement: Additional file 8: Figure S6. — Un-cropped images and additional views from the cell shown in Fig. 6. (A, B) Slices 000 and 220 in xy orientation, with the intracellular plasma membrane-connected compartment (IPMC) segmented in green and the cell surface plasma membrane in yellow. (C–E) xz side view as in Fig. 6k. The positions of section 000 and section 220 are indicated by dashed lines. (D) and (E) show the same view as in (C) with the cell surface (yellow) transparent (D) or opaque (E). (F, G) Uncropped xy view as in Fig. 6l–n, with cell surface virus particles only (cyan, F) or with the cell surface (transparent yellow, G). (H–J) yz side views, representation as in (C–E). Note the clustering of cell surface virus particles (cyan) above IPMC exit sites. (TIF 15003 kb) [file 12915_2016_272_MOESM8_ESM.tif]
